# Supplementary material for: Accelerated knowledge discovery from omics data by optimal experimental design
Source: Nat Commun. 2020 Oct 6;11:5026. doi: 10.1038/s41467-020-18785-y (PMC7538421; doi:10.1038/s41467-020-18785-y)
Supplement: Supplementary file 1 — Supplementary Information [file 41467_2020_18785_MOESM1_ESM.pdf]

Accelerated knowledge discovery from omics data by optimal experimental design

Wang et al

Supplementary Information

Supplementary Methods

1. The OPEX Framework

1.1 Overview

We have designed the Optimal Experimental Design Framework (OPEX) to identify optimal set of transcriptomic experiments for maximizing prediction power in unobserved culture conditions in three steps (Fig. 1, steps 1-3). In the first step, we use the available transcriptomic data to *Build Predictive Model* of gene expression using culture condition as the model input. In the second step, we *Calculate Utility Scores* for unobserved culture conditions using the predictive model from the first step. In the third step, we *Select Optimal Conditions* amongst all unobserved culture conditions given their utility scores from the second step. In its general form, OPEX is the following optimization problem:

$$X_\mathrm{s} = \underset{X}{\mathrm{ArgMax}}\ batch\_optimality(X, b, X_\mathrm{o}, Y_\mathrm{o}) \tag{1}$$

where the matrix X_s denotes the culture conditions for the next batch of experiments, the matrix X_o denotes the culture conditions for the observed experiments (with each row of the matrix being an experiment), matrix Y_o contains the gene expression profiles that map to the corresponding experiments of X_o and scalar b denotes the batch size (i.e. number of conditions to run for the next batch). The optimality of a batch of candidate conditions in matrix X is determined using the function $batch\_optimality$ and the optimal batch is returned by $\underset{X}{\mathrm{ArgMax}}$.

1.2 General Mathematical Formulation

The following describes the three-step algorithm of OPEX algorithm for finding X_s. The modular design of the OPEX algorithm (Algorithm 1) allows different methods to be used in each step.

Algorithm 2. OPEX algorithm to identify the optimal set of experiments at each round.

Inputs: $\{X_{\mathrm{o}}, Y_{\mathrm{o}}, X_{\mathrm{u}}, b\}$, where the observed conditions are characterized by matrix X_{o}, their corresponding gene expression profiles are in matrix Y_o, the unobserved conditions are characterized by matrix X_{u}, and the batch size (i.e. the number of conditions to perform next) is denoted by scalar b.

Output: $\{X_{\mathrm{o}}, Y_{\mathrm{o}}\}$, where X_o and Y_o include the original input and the newly collected data.

for t = 1 **to** T **do** // Run OPEX for T rounds
    for j = 1 **to** m **do** // Run OPEX for all m genes
        $\mathbf{y}_{\mathrm{o}} \leftarrow Y_{\mathrm{o}}^{\mathrm{j}}$ // Select the jth gene
        $Model \leftarrow build\_predictive\_model(X_{\mathrm{o},}\, \mathbf{y}_{\mathrm{o}})$ // OPEX Step 1 (see section 1.2.1)
        $\boldsymbol{s}_{\mathbf{u}} \leftarrow calculate\_utility\_scores(Model, X_{\mathrm{u}})$ // OPEX Step 2 (see section 1.2.2)
        $X_{\mathrm{s}}^{\mathrm{j}} \leftarrow select\_optimal\_conditions(X_{\mathrm{u}}, \boldsymbol{s}_{\mathrm{u}}, b)$ // OPEX Step 3 (see section 1.2.3)
    end for
    $X_{\mathrm{new}} \leftarrow select\_most\_frequent\_conditions(X_{\mathrm{s}}^{1}, X_{\mathrm{s}}^{2}, \ldots, X_{\mathrm{s}}^{\mathrm{m}})$ // (see section 1.2.4)
    $Y_{\mathrm{new}} \leftarrow run\_experiment(X_{\mathrm{new}})$ // Wet lab experiments
    $(X_{\mathrm{o},}\, Y_{\mathrm{o}}, X_{\mathrm{u}}) \leftarrow update\_data(X_{\mathrm{o},}\, Y_{\mathrm{o}}, X_{\mathrm{new}}, Y_{\mathrm{new}})$ // Update datasets
end for
return $(X_{\mathrm{o}}, Y_{\mathrm{o}})$

The vector $\boldsymbol{s}_{\mathrm{u}}$ contains n_{u} real valued utility scores, one for each unobserved condition that is encoded by a corresponding row of X_{u}.

Modeling Culture Conditions. The $n_{\mathrm{o}} \times m$ matrix X_{o}, characterizes n_{o} observed culture conditions in its rows using m independent variables as columns. Examples of independent variables include the antibiotic concentration and temperature. Matrices X_{u} and X_{s} characterize n_{u} unobserved and n_{s} selected culture conditions respectively in the same way both with m columns. Note that OPEX allows independent variables to be modeled as binary or real values.

Modeling Gene Expression Profiles. The $n_{\mathrm{o}} \times d$ matrix Y_{o}, contains n_{o} gene expression profiles in its rows for d genes modeled by its columns. Gene expression profiles of OPEX are modeled as real values.

Next, we describe the methods used in our implementation and results.

1.2.1 Build Predictive Model

For each gene of *E. coli*, we used Gaussian Processes (GP) to build a predictive model (i.e. $Model(x)$) to predict the expression level of the gene under a culture condition characterized by a horizontal vector $\boldsymbol{x}$. In our real-data results, $\boldsymbol{x}$ is a 14-bit binary vector representing presence/absence of ten biocides and four antibiotics which characterize a given culture condition. In our synthetic-data results, $\boldsymbol{x}$ is a real valued vector with length 2 characterizing the concentrations of one biocide and one antibiotic in each culture condition.

The observed expression y of a gene is a population average of the expression levels of the gene in multiple cells. According to the central limit theorem, the observed expression level of a gene under a culture condition follows a Gaussian distribution. Given a new unobserved condition ($\boldsymbol{x}^{new}$), the predicted mean of y^{new}, $E(y^{\mathrm{new}})$, and the predicted variance of y^{new}, σ^2_{new}, are computed by equation 2 and 3, respectively:

$$E(y^{\mathrm{new}}) = \boldsymbol{k}^{\mathrm{new}} K^{-1} \boldsymbol{y}_{\mathrm{o}}^{\mathrm{T}} \quad (2)$$

$$\sigma^2_{\mathrm{new}} = k(\boldsymbol{x}^{\mathrm{new}}, \boldsymbol{x}^{\mathrm{new}}) - \boldsymbol{k}^{\mathrm{new}} K^{-\mathbf{1}} \boldsymbol{k}^{\mathrm{new}^{\mathrm{T}}} \quad (3)$$

$$Model(x^{\mathrm{new}}) \sim \mathcal{N}(E(y^{\mathrm{new}}), \sigma^2_{\mathrm{new}}) \quad (4)$$

where, $\boldsymbol{k}^{\mathrm{new}} = [k(\boldsymbol{x}^{\mathrm{new}}, \boldsymbol{x}_{\mathrm{o}}^1), k(\boldsymbol{x}^{\mathrm{new}}, \boldsymbol{x}_{\mathrm{o}}^2), \dots, k(\boldsymbol{x}^{\mathrm{new}}, \boldsymbol{x}_{\mathrm{o}}^n)]$ is a vector, representing the correlation between y^{new} and the gene expression for *n* observed culture conditions, ($\boldsymbol{y}_o = [y^1, y^2, \dots, y^n]$). The kernel function, $k(\boldsymbol{x}^i, \boldsymbol{x}^j)$, models the correlation between y^i and y^j, using a squared exponential (SE) function:

$$k(\boldsymbol{x}^i, \boldsymbol{x}^j) = \sigma \exp\left(-\sum_{p=1}^{14} \frac{\left({x_p}^i - {x_p}^j\right)^2}{\theta_p}\right) \quad (5)$$

The covariance matrix $K = \begin{bmatrix} \boldsymbol{k}^1 \\ \boldsymbol{k}^2 \\ \vdots \\ \boldsymbol{k}^n \end{bmatrix}$ represents all pairwise correlations for the given gene. The parameter σ represents the amplitude of overall correlation along all dimensions in the SE kernel while parameter θ_p is used for automatic relevance determination [1]. A larger value of θ_p represents a smaller influence of the pth independent variable of a culture condition on the gene expression. These parameters are learned by maximizing the marginal likelihood of the observed data given the parameters. For a detailed derivation of the equations related to GP, see [1].

Given the selection of GP as our model in this work (from equations (1-4)), for each gene the trained $Model(x^{\text{new}})$ is well defined by $\boldsymbol{k}^{\text{new}}, K$ and $\boldsymbol{y}_{\mathbf{o}}$ which are used to predict the gene expression using $E(y^{\text{new}})$ from equation 2 and to calculate utility scores as described next.

1.2.2 Calculate Utility Scores

We evaluated OPEX using three different utility score calculation methods described here. The utility score s_{new} for a new unobserved condition is calculated using the utility function. The utility scores of all unobserved conditions are represented by vector $\boldsymbol{s}_{\mathbf{u}} = [s_{\text{u}}^1, s_{\text{u}}^2, \ldots, s_{\text{u}}^m]$, where s_{u}^j is the utility score of the jth unobserved condition for a given gene.

Entropy (EN). The gene expression level predicted by a trained GP model follows a Gaussian distribution, therefore the distribution of y^{new} is fully defined by (y^{new}) and σ_{new}^2. Following the definition of entropy (equation 5), we can easily derive the entropy of the gene expression (equation 6) by plugging into the probability density function of y^{new}.

$$H(y^{\text{new}}) = \int p(y^{\text{new}})\, ln\big(p(y^{\text{new}})\big)\, dy^{\text{new}} \quad (6)$$

$$H(y^{\text{new}}) = 0.5\, log(2\pi e\, \sigma_{\text{new}}^2) \quad (7)$$

Equation 6 shows that entropy is a monotonic function of the variance of y^{new}. Intuitively, a condition with a high σ_{new}^2 has a high utility explained by the uncertainty of GP model about this new condition. Plugging equation 3 into equation 6, we get equation 8 to calculate the entropy.

$$s_{\text{new}} = o.5\log(2\pi e(k(\boldsymbol{x}^{\text{new}}, \boldsymbol{x}^{\text{new}}) - \boldsymbol{k}^{\text{new}} K^{-\mathbf{1}} \boldsymbol{k}^{\text{new}^{\text{T}}})) \quad (8)$$

where $\boldsymbol{k}^{new}$ and K are the same as in equation 2.

Mutual Information (MI). In the setting of MI, the idea is to select the most representative culture condition amongst all possible unobserved culture conditions. The representativeness of n observed culture conditions is quantified by the mutual information (MI) between the observed and the unobserved gene expression $\boldsymbol{y_o}$ and $\boldsymbol{y_u}$. The MI can be calculated by equation 9 where $H(\boldsymbol{y_u})$ represents the entropy of the gene expression under all unobserved culture conditions and $H(\boldsymbol{y_u}|\boldsymbol{y_o})$ represents the entropy of the gene expression under all unobserved culture conditions given the gene expression under the observed culture conditions.

$$MI\ (\boldsymbol{y}_{\text{o}}, \boldsymbol{y}_{\text{u}}) = H(\boldsymbol{y}_{\text{u}}) - H(\boldsymbol{y}_{\text{u}}|\boldsymbol{y}_{\text{o}}) \quad (9)$$

Given a set of observed conditions X_{o}, the utility score for each unobserved culture condition x_{new} is equivalent to the increase in $MI\ (\boldsymbol{y}_{\text{o}}, \boldsymbol{y}_{\text{u}})$ if we move the x_{new} to the set of observed conditions. Plugging equation 7 into equation 9, we can derive the utility of a new condition using the following equation (see the section 4.1 of reference [2] for detailed derivation):

$$s_{\text{new}} = MI\ (\boldsymbol{y}_{\{\text{o+new}\}}, \boldsymbol{y}_{\{\text{u-new}\}}) - MI\ (\boldsymbol{y}_{\text{o}}, \boldsymbol{y}_{\text{u}}) = \frac{\sigma_{\text{new}}^2 - \boldsymbol{k}^{\text{new}}\ K^{-1}\ \boldsymbol{k}^{\text{new}^T}}{\sigma_{\text{new}}^2 - \boldsymbol{k}_{\{\text{u-new}\}}^{\text{new}}\ K_{\{\text{u-new}\}}^{-1}\ \boldsymbol{k}_{\{\text{u-new}\}}^{\text{new}\ T}} \quad (10)$$

where $\boldsymbol{y}_{\{\text{o+new}\}}$ is the gene expression in all the observed conditions and the new condition; $\boldsymbol{y}_{\{\text{u-new}\}}$ is the gene expression in all unobserved conditions except the new condition; $\boldsymbol{k}^{\text{new}}$ and K are the same as in equation 2; $\boldsymbol{k}_{\{\text{u-new}\}}^{\text{new}}$ is a vector composed of the covariance between the new condition and all other unobserved condition. $K_{\{\text{u-new}\}}$ is the pairwise covariance between all unobserved conditions except the new condition.

Covariance (COV). Similar to MI, the covariance captures how the observed culture conditions relate to the unobserved ones. One sequential design implementation is to select the culture condition which provides the highest increase in covariance between the observed datapoints and unobserved datapoints

[3]. The covariance matrix can be calculated by the following equation for a given gene.

$$COV_{\mathrm{u,o}} = K_{\mathrm{u,o}} K_{\mathrm{o,o}}^{-1} K_{\mathrm{o,u}} \tag{11}$$

$K_{\mathrm{u,o}}$ is a covariance matrix composed of the pairwise correlation between the unobserved conditions and the observed conditions. Each entry in the matrix is calculated by the kernel function of the GP. $K_{\mathrm{o,o}}$ a covariance matrix composed of the pairwise correlation between the observed conditions. The covariance utility function is equal to the increment in the trace of the covariance matrix calculated by the following equation:

$$s_{\mathrm{new}} = trace(COV_{\{\mathrm{u-new}\},\{\mathrm{o+new}\}}) - trace(COV_{\mathrm{u,o}}) \tag{12}$$

where $COV_{\{\mathrm{u-new}\},\{\mathrm{o+new}\}}$ is the same as $COV_{\mathrm{u,o}}$ except that a given unobserved condition $\boldsymbol{x}^{\mathrm{new}}$ is removed from the set of unobserved and added to observed conditions for a given gene.

1.2.3 Select Optimal Conditions for each gene

Following the general optimization equation (1) and using the terminology above, the next condition to select is the one that has the maximum utility score according the jth gene is:

$$\boldsymbol{X}_s^j = \underset{x_{\mathbf{new}}}{\mathbf{ArgMax}}\ s_{\mathrm{new}} \tag{13}$$

where s_{new} is the utility score corresponding a given unobserved x_{new} condition calculated by one of equations 8, 10, and 12, depending on the utility function used. For example, when we use mutual information (i.e. equation (10)) as the utility function, the most informative condition for j^{th} gene is selected by solving the following OPEX optimization problem:

$$\boldsymbol{X}_{\mathrm{s}}^j = \underset{x_{new}}{\mathbf{ArgMax}}\ \frac{\sigma_{\mathrm{new}}^2 - \boldsymbol{k}^{\mathrm{new}}\, K^{-1}\, \boldsymbol{k}^{\mathrm{new}^T}}{\sigma_{\mathrm{new}}^2 - \boldsymbol{k}_{\{\mathrm{u-new}\}}^{\mathrm{new}}\, K_{\{\mathrm{u-new}\}}^{-1}\, \boldsymbol{k}_{\{\mathrm{u-new}\}}^{\mathrm{new}\ T}} \tag{14}$$

Subject to $\sum_{i=1}^{10} x_i^{\mathrm{new}} = 1, \sum_{j=11}^{14} x_j^{\mathrm{new}} = 1, x_j \in \{0, 1\}$

where $\boldsymbol{x}_{\text{new}}$ is a 14-bit binary vector representing presence/absence of ten biocides and four antibiotics for a culture condition.

Finally, $[\boldsymbol{X}_{\mathbf{s}}^{1}, \boldsymbol{X}_{\mathbf{s}}^{2}, \ldots \boldsymbol{X}_{\mathbf{s}}^{m}]$, where m is the number of genes. With the optimal unobserved conditions selected for each gene, we count the frequency of each selected batch and select the most frequent one from

For a larger batch size (i.e. $b > 1$), the next condition in the batch was selected by greedy, constrained or adaptive sampling, as described in the respective results. In **greedy sampling**, the conditions are ranked based on their utility scores. The top b conditions with highest utility scores are selected for the next batch. In **constrained sampling,** the condition with the highest utility score is selected and added to the batch. Then we iterate through the remaining conditions ordered by their utility scores and calculate their Euclidean distance to selected items in the batch. Conditions with a minimum distance (based on a predefined threshold) are added until the batch-size limit b is reached. Finally, in **adaptive sampling**, the condition with the highest utility score is selected and added to the batch. Then, the predicted gene expression profile of the newly selected condition is considered as observed leading into the newly trained model and updated utility scores. The condition with the highest updated utility score is then added to the batch. This process is repeated until the batch-size limit b is reached.

1.2.4 Select Optimal Conditions for most of genes

With the optimal unobserved conditions selected for each gene, we count the frequency of each selected batch and select the most frequent one from $[\boldsymbol{X}_{\mathbf{s}}^{1}, \boldsymbol{X}_{\mathbf{s}}^{2}, \ldots \boldsymbol{X}_{\mathbf{s}}^{m}]$, where m is the number of genes.

2. Computational Methods

2.1 Alternative Optimal Experimental Design Methods

For benchmarking, we used three other optimal experimental design approaches, query by the committee using different types of models [4-5], query by committee using bootstrapping [6], and D-optimal experimental design [7]. Compared to OPEX, all approaches differ in the utility function used while the

last approach also employs a different predictive model.

Query by Committee Using Different Types of Models. We used feedforward neural network (FNN), linear regression, Gaussian process and Support Vector Regression (SVR). For training an FNN and SVR, we used the packages, neuralnet and e1071, respectively. The number of hidden nodes of FNN and the two hyperparameters of SVR were optimized by grid search. In each iteration, the condition with the highest disagreement amongst different models (i.e. highest variance) was selected for the next iteration. When generating a learning curve, GP model was used.

Query by Committee Using Bootstrapping. Here we used one type of model (GP), but changed the training set using bootstrapping to build a committee of four GP models. Likewise, the condition with the highest disagreement amongst different models (i.e. highest variance) was selected for the next iteration. When generating the learning curve, the GP model was trained without bootstrapping.

D-optimal Experimental Design. Here, we used a linear model to predict gene expression (linear models were trained by the built-in implementation for linear regression in R). The condition that increased the determinant of information matrix (X^TX) to the most extent was selected at each iteration. X is a matrix consisting of the vectors each of which represents a culture condition.

2.2 Expert Sampling

We designed three strategies for expert sampling by consulting one chemist and three biologists to evaluate the effectiveness of OPEX compared to human experts. These are:

First strategy: Structural similarity. The first strategy relied on comparing the pairwise structural similarity among 10 biocides and 4 antibiotics. The least similar culture condition was selected in each iteration. Specifically, a 1024-bit topological fingerprint was generated for each chemical using the Python package, rdkit. The pairwise Tanimoto similarity among the biocides and antibiotics were calculated [8]. The similarity between the two culture conditions was defined as the sum of the similarity between the two biocides and that between the two antibiotics. When exploring the space defined by the biocides and antibiotics, we looked up the similarity of each unobserved culture condition to all the

observed culture conditions and picked the least similar one.

Second strategy: Mechanism of action. The second strategy was based on similarity in the mechanism of action of each antibiotic and biocide. In each iteration, we sampled the antibiotic and biocide that were most different from the observed ones. For the mechanism of action of each antibiotic and biocide, see Supplementary Table 1.

Third strategy: Effect size. For the third strategy, three experts first ordered the four antibiotics based on their expected dominant impacts on transcription in the central dogma of molecular biology, Ampicillin < Norfloxacin < Kanamycin < Rifampicin. Rifampicin is known to inhibit RNA polymerase hence directly impacting transcription [9]. Kanamycin interferes with translation hence indirectly impacting transcription through transcription factors [10]. Norfloxacin and Ampicillin are known to impact DNA replication and cell wall hence ordered last with respect to their impact on overall transcription profile [11-12]. If an antibiotic is dominant, the choice of biocide would be expected to have a smaller impact on gene expression of *E. coli*. We rationalized that if we have the gene expression under a culture condition that has a more dominant antibiotic, we are likely to do a good prediction for the culture conditions that have the same antibiotic but different biocides. Based on such reasoning, we grouped the unobserved culture conditions into four groups based on the antibiotic. The group that has a less dominant antibiotic was sampled before the group that has a more dominant antibiotic. In each group, we split the culture conditions into 5 buckets based on the mechanism of each biocide. Among the 5 buckets, we randomly selected one in each iteration while making sure two consecutive datapoints were not from the same bucket.

2.3 Random Sampling

For random sampling, we randomly selected a datapoint (an experimental condition in our setting) from all the unobserved datapoints as the next datapoint to collect. The default random function in R was used.

2.4 Exploration-Exploitation Tradeoff

The calculated utility scores have a potential myopic bias, therefore relying on them for selecting the

next batch of experiments (i.e. exploitation of the model) can lead to overfitting. To avoid this, a portion of the selected conditions for the next batch can be selected randomly (i.e. exploration of the sample space). The exploration-exploitation trade-off is fundamental in optimal experimental design [2]. Exploration refers to switching to a strategy different from the predefined strategy based on one of the utility functions. The role of exploration is similar to simulated annealing [14]. Exploitation means exploiting the information learned from the collected data and selecting the next datapoint based on the prediction of a model trained on the collected data. We used exploration frequency to control the tradeoff. The effect of the exploration frequency parameter was evaluated on synthetic datasets and the RNA-Seq dataset.

2.5 Synthetic and Real RNA-Seq Datasets

OPEX was evaluated on a synthetic dataset and real RNA-Seq datasets.

2.5.1 Synthetic Datasets Generation. The observed response of a biological system to culture condition perturbations, can be modeled in a multi-dimensional space using a dependent response variable and a set of independent variables. Such models are usually non-smooth and rugged with a few peaks or valleys in the space. To simulate such rugged spaces, the sum of 300 bi-variate normal distributions with different means and a variance of 1 were superimposed and taken to the power of 2, 3, 4, 5, 6, 7, and 8, generating seven datasets, with varying skewness. The two dimensions of the bi-variate normal distributions simulated various concentrations of an antibiotic and a biocide and the value of the superimposed normal distributions represents the gene expression in a culture condition defined by the antibiotic and biocide. The impact of noise; a property of biological data; was also evaluated by adding white noise with levels proportional to the gene expression level for each datapoint.

2.5.2 RNA-Seq Dataset

We measured the gene expression profiles of *E. coli* under 45 culture conditions defined by 4 antibiotics and 40 combinations of 10 biocides and 4 antibiotics, and an untreated control. Out of all the genes of *E. coli*, 1,123 genes had a count per million (CPM) larger than 100 in at least half of the samples. The fold changes of the genes that have a CPM less than 100 in at least half of the samples are expected to

be sensitive to the sequencing depth [13]. To exclude the effect of those genes, we tested OPEX only using the 1,123 genes. We also tested OPEX and variations of OPEX on all the 4,391 genes, which resulted in similar results (Section 2.5).

2.5.3 Validating OPEX on Synthetic Datasets

When validating the performance of OPEX on the synthetic datasets, we first randomly split a synthetic dataset into three datasets, a training dataset, a pool of candidate conditions, and a benchmark set for evaluating the prediction performance of a trained predictive model. Then we evaluated OPEX in 30 iterations. In each iteration, we trained a GP model using the training dataset, calculated the utility score of each candidate condition that remained in the pool and selected a batch of conditions for adding to the training set. Finally, we evaluated the predictive performance by the mean absolute error of predictions on the benchmark set. After running OPEX for 30 iterations, we visualized the prediction accuracy at each iteration as a learning curve and compare the learning curve of OPEX and that of the baseline. When selecting a batch of conditions in a batch, we tested three approaches as outlined in section 1.2.3. Random sampling was used as the baseline for evaluating the performance of OPEX.

2.6 Validating OPEX on an RNA-Seq Dataset

Since the RNA-Seq dataset does not have as many records as in the synthetic datasets, we slightly changed our validation method for it. We first split the whole dataset into two parts instead of three. One part served as the starting point of the training set. The other part served both as the pool of culture conditions for selection, and as the benchmark dataset. The initial training set consisted of 15 randomly selected culture conditions where each antibiotic and biocide were selected at least once. In each iteration, we trained a GP using the current training set, selected a candidate culture condition from the pool and moved it to the training dataset, then evaluated the prediction performance of the retrained GP on the benchmark set. Note that the size of the benchmark was reduced at each iteration. We run 50 times the whole process with a different random seed at each time.

Two types of methods were used for comparison, random sampling and expert sampling (for details, see the section entitled Expert Sampling). Each sampling method was evaluated against random sampling

using the MAE of gene expression predictions in a given iteration.

2.7 Cluster Analysis on 40 Culture Conditions

We ran Principal Component Analysis (PCA) [15] and t-Distributed Stochastic Neighbor Embedding (t-SNE) [16] on the gene expression profiles of all 40 conditions using prcomp and Rtsne respectively in the R programming language, and projected the first two dimensions.

Hierarchical clustering was also performed on all 40 gene expression profiles (Fig. 3B). First, gene set enrichment analysis (GSEA) was conducted on all transcriptomic datasets using the GSEA v3.0 software with the default parameters. Only dysregulated GO-BP terms (i.e. gene ontology terms relating to biological processes) with normalized p-values <0.05, were selected for further analysis. The hierarchical agglomerative clustering of DEGs and BPs of all antibiotics and biocide-antibiotic pairs used the nearest point algorithm (the built-in function in R for hierarchical clustering was used).

2.8 OPEX Accelerates Knowledge Discovery

To test whether OPEX can accelerate knowledge discovery (Fig. 3C), we started with gene expression profiles of 15 culture conditions and predicted the gene expression profiles of the 30 unobserved culture conditions. With 15 measured and 30 predicted gene expression profiles, we run hierarchical clustering and evaluated the accuracy of the membership of the 30 conditions. Likewise, we run OPEX for 30 iterations and evaluated the accuracy at each iteration.

3. Experimental Methods

3.1 Antimicrobials

10 biocides and 4 antibiotics were used in this study. Biocides were selected based on their widespread use in hospitals and households [17], and antibiotics were selected based on their unique cellular targets (Supplementary table 1). Biocides used in this work were: benzalkonium chloride (MP Biomedicals), hydrogen peroxide (Macron), peracetic acid (Sigma Aldrich), sodium hypochlorite (Sigma Aldrich), glutaraldehyde (Amresco), chlorhexidine (Sigma-Aldrich), chlorhexidine gluconate (Spectrum),

povidone iodine (Sigma-Aldrich) and chlorophene (Sigma-Aldrich). Four antibiotics used for this work were: ampicillin (Roche-Diagnostics), kanamycin (Acros Organics), norfloxacin (Sigma-Aldrich), and rifampicin (Cayman Chemical). Antibiotic stocks were stored at -800℃ until used and biocides were prepared on the day of experiment. Working concentrations of biocides and antibiotics were: benzalkonium chloride (3.63 mg/L), isopropanol (2.7% v/v), ethanol (2.8% v/v), hydrogen peroxide (272 µM), peracetic acid (9 µM), sodium hypochlorite (3.64 µM), glutaraldehyde (29 µM), chlorophene (0.25 mM), chlorhexidine (1.48 µM), povidone iodine (12.5 µg/mL), ampicillin (0.80 µg/mL), kanamycin (4.0 µg/mL), norfloxacin (0.024 µg/mL), rifampicin (8.0 µg/mL).

3.2 Strains and Culture Conditions

Escherichia coli MG1655 was used in all experiments, excluding the experiments performed to validate the genes involved in cross-protection and cross-vulnerability where wild type Keio strain BW25113 and its derivative single gene knock out (KO) strains [18] were used. Since, KO strains had kanamycin resistance gene, which might influence the validation experiment, it was removed by a method described elsewhere [19].

Supplementary Results

4.1 Computational Results on Synthetic Datasets.

4.1.1 Synthetic Datasets

Seven datasets whose skewness in the distribution of the output varies from 1.17 to 7.86 were generated. In a highly skewed dataset, in most cases the output was close to zero and only several sharp peaks exist in the space. See Supplementary Figure 1, for a visualization of the datasets, distribution of the output of each dataset and statistics of the datasets. The data for the synthetic datasets is in Supplementary Data 2.

4.1.2 The Performance of OPEX on Synthetic Datasets

We evaluated the performance of OPEX using seven synthetic datasets with respect to five factors including: skewness in the distribution of the output, the noise level in the measured output, frequency of exploration, initial dataset size, and batch size. We did not find such a systematic analysis of these factors in another study.

The Effect of Skewness and Noise. Interestingly, the advantage of OPEX advantage concerning the baseline was inversely proportional to the skewness of the dataset (p-value $< 10^{-3}$ by t-test; Supplementary Figure 2A). OPEX was found to be robust when noise is present in the training set, outperforming the baseline even at very high noise levels (for the entropy utility function, 16% better than the baseline at 90% white noise on the 1st synthetic dataset, p-value $< 10^{-6}$ by t-test; Supplementary Figure 2B and Supplementary Figure 3).

The Effect of Exploration. We increased the level of exploration in OPEX by switching to random sampling in some iterations and did not observe improvement in the OPEX's performance on the synthetic datasets (Supplementary Figure 2C and Supplementary Figure 4).

The Effect of Initial Dataset Size. Regarding the initial dataset size, there was a window where the benefit is maximized (Supplementary Figure 2D), and that window varied given the dataset skewness

(Supplementary Figure 5). When the dataset size was too small, the benefit of OPEX methods was generally limited until more samples were collected. When enough information is not initially available, we don't expect OPEX to effectively drive experimentations. Similarly, when the initial dataset size was large relative to the size of the experimental space, the experimental space has been largely explored. Therefore, increasing the dataset size with more experiments does not impact the information content of the dataset regardless of the underlying method sampling method (e.g. OPEX vs. Random sampling).

The Effect of Batch Size. The batch size (i.e. the number of experiments performed per iteration), also had a substantial effect on the efficiency of a method for larger batch sizes (batch size ≥16). The best and more robust performance was observed when we ran OPEX with adaptive sampling (Supplementary Figure 6A). OPEX with constrained sampling did not work on large batch sizes. Finally, for OPEX with greedy sampling, smaller batch sizes generated better performance, as expected. There was a 28% average performance gain for OPEX using entropy on batch size of 2 versus batch size of 32 (p-value = 1×10^{-8}; Supplementary Figure 6C). This can be explained by the redundancy among points close to each other on the rank list. More skewed datasets were less sensitive to batch sizes (Supplementary Figure 7 and Supplementary Figure 8).

4.2 Computational Results on the RNA-Seq Dataset

4.2.1 OPEX Performance on the Biocide-Antibiotic Transcriptional Profiling

OPEX with entropy as the utility function, outperformed expert sampling and random sampling significantly for exploring the interaction between biocides and antibiotics (Supplementary Figure 9). The gap between the learning curves of OPEX and random sampling kept expanding until 23 more datapoints were collected and the MAE achieved by OPEX was 22% smaller than random sampling at that point. To reach the same prediction accuracy by random sampling, OPEX needed 50% fewer datapoints.

Surprisingly, the performances of the three expert sampling strategies was worse than that of random sampling. Among the three expert sampling approaches, the one based on the chemical structure of antibiotics and biocides is slightly better than the other two (Supplementary Figure 10). When adding

more and more exploration, the performances of expert sampling strategies became close to that of random sampling but never surpassed (Supplementary Figure 10).

4.2.2 Retrospective Analysis of the OPEX Strategy

To analyze the effectiveness of OPEX in exploring the space of unexplored culture conditions, we plotted the distance between the gene expression profiles of consecutively selected datapoints (the consecutive distance) over 30 iterations. Not surprisingly, the consecutive distance fluctuated, and no pattern was observed in the case of random sampling (Fig. 2D). However, the consecutive distance for OPEX with an even tradeoff between exploration and exploitation increased gradually in the first 10 iterations (p-value = 0.05), and finally kept decreasing (p-value < 10^{-6}, Fig. 2E), indicating that OPEX can capture the similarity of gene expression profiles under different culture conditions. This, reveals the underlying strategy of OPEX in progressive exploration of the condition space, first at a coarse granularity and then on a finer granularity, which was confirmed by the fact that the distance in the first 15 iterations was above the median distance of all the 30 iterations, and the distance in the latter 15 iterations fell below the median. In more detail, the distance between adjacent points in the gene expression space increased in the first 10 iterations and decreased afterwards, showing that OPEX explored the space on an increasingly higher level of granularity and then decreased the level of granularity. The impact of exploration percentage used by OPEX on the sampling strategy, is illustrated in Supplementary Figure 11.

In the case of expert sampling based on structure similarity, the consecutive distance first fluctuated and increased sharply at the end (Supplementary Figure 12A). For the other two expert sampling approaches, the consecutive distance in the first 10 iterations was flat and then increased slightly and finally decreased slightly (Supplementary Figure 12B-C). Not surprisingly, it gets closer to the random sampling curve as additional exploration percentage is added (Supplementary Figure 12D-I).

4.2.3 Sensitivity Analysis of the OPEX Method

Here, we investigated the impact of the exploration frequency, skewness and noise level on the performance of OPEX evaluated using RNA-Seq dataset.

The Effect of Exploration. When the space to explore is of low complexity (e.g. convex fitness functions, few parameters/dimensions) following a single sampling strategy with zero percentage of exploration used by OPEX is sufficient, as it was the case with the synthetic dataset (Supplementary Figure 4). However, for a complex space as in the case of RNA-Seq data with 14 independent variables and thousands of genes to predict, OPEX with zero exploration can overfit (Supplementary Figure 11A-B). Such model behavior was observed on expert sampling (Supplementary Figure 12), and when entropy or mutual information were used as the OPEX utility functions. OPEX tended to take a greedy approach with a bigger step (i.e. larger distance between gene expression profile of subsequently selected conditions) at the beginning for exploring the space and a gradual reduction of the step sizes later on (Supplementary Figure 11A-B). Conversely, OPEX with an even tradeoff between exploration and exploitation showed a smoother sampling strategy with a gradual increase of step size in the beginning and a decrease in later iterations (Fig. 2D and Supplementary Figure 11C-D).

We evaluated the performance of OPEX with various levels of exploration. The learning curve of OPEX was sharper at the beginning when less exploration was used (Supplementary Figure 13A-B). When zero percentage of exploration was applied, the learning curve started to go up at around iteration 16. Our validation indicated that this sharp increase after iteration 16 was partly due to an outlier dominating the errors as the number of remaining conditions decreased (Supplementary Figure 14B-F). This outlier condition was peracetic acid + kanamycin.

We analyzed the diversity of the selected condition at each iteration among all the OPEX runs. Shannon index was used to quantify the diversity of the sampled conditions at each iteration (Supplementary Figure 15A). The diversity of the selected condition at each iteration among all the OPEX runs for OPEX were very low compared to that of random sampling, which is indicative of the tendency of OPEX in selecting particular conditions at each iteration, suggesting that OPEX tended to sample this outlier condition (i.e. peracetic acid + kanamycin) regardless of the starting training datapoints. We confirmed this by visualizing the distribution of the culture conditions selected by OPEX at the last two iterations. At the 27th and 28th iteration, OPEX chose the peracetic acid + kanamycin condition 33 times among the 50 OPEX runs (Supplementary Figure 15B-C). Note that for the initial dataset (i.e. 15 randomly selected conditions) the peracetic acid + kanamycin condition was only selected in 11 OPEX runs (amongst 50

total runs). The expected number of conditions that include a specific condition in 50 OPEX runs is 11.2. Thus, OPEX chose to sample peracetic acid + kanamycin condition at the end in 84% of the 39 OPEX runs (39=50-11). When adding 50% of exploration, the diversity was increased (Supplementary Figure 15D-E) and the performance of OPEX was optimal (Supplementary Figure 14). Similarly, the diversity can be increased by adding more exploration in the case of expert sampling (Supplementary Figure 15F), but since the sampling strategy of expert sampling was not effective, the performance could not surpass that of random sampling (Supplementary Figure 10).

The Effect Noise and Skewness. Finally, we visualized the performance of OPEX with respect to the noise level in the measured gene expression and skewness in the distribution of the expression levels for the 1,123 genes. OPEX performed well regardless of the noise level on most of the genes (969 out 1,123, 86%, Supplementary Figure 16). The performance of OPEX increased slightly with skewness, which is different from what we observed on the synthetic datasets. This difference may be due to other factors impacting the topology of the space (e.g. number of genes and independent variables). We also performed gene ontology (GO) enrichment analysis on the list of 969 genes using DAVID [20]. The resulting enriched biological process GO terms were related to translation, glycolytic processes, cell division, peptidoglycan biosynthetic processes, and regulation of cell shape (with a threshold for the adjusted p-value of 0.01). No enriched biological process GO terms were observed for the 154 genes (14%) for which OPEX did not outperform the expert sampling.

4.2.4. Peracetic Acid and Kanamycin Condition as an Outlier

We investigated further why OPEX deprioritizes the selection of peracetic acid + kanamycin condition until later iterations while having a poor performance for predicting its gene expression profile. We hypothesized that the GP model predicted a gene expression profile for this condition that was similar to the ones in the training dataset. Thus, we visualized the predicted gene expression profile of those conditions with the measured gene expression profile for other conditions in 2d space by t-SNE (Supplementary Figure 17). The predicted gene expression was close to other conditions in which peracetic acid or kanamycin was present. Since the gene expression was predicted based on the culture conditions, it is reasonable that the model made such a prediction. However, the true gene expression

under the peracetic acid + kanamycin condition was close to another antibiotic (the cluster in the top right of Fig. 3A, the green cluster in Supplementary Figure 18), which suggests that the model could not determine the gene expression of that condition based on the antibiotic and biocide used.

4.2.5 Validating OPEX on all 4,391 Genes

We have shown the superior performance OPEX when evaluated using 1,123 genes which meet minimum sequencing coverage criteria (count per million >100, as described under RNA-Seq data analysis in Methods section of the main manuscript), following recommended guideline for RNA-Seq analysis. This raises a question on whether OPEX also performs well in the case that the set of inactive genes are unknown beforehand. Therefore, we also assessed the performance gain of OPEX compared to random sampling without removing any genes (i.e. used all 4,391 genes), and achieved similar results. OPEX needed approximately 40% datapoints to reach the same accuracy compared to random sampling (See Supplementary Table 2 and Supplementary Figure 19), similar to results of Fig. 2B where only 1,123 were used. We compared OPEX runs that consider all 4,391 genes versus OPEX runs that rely on the 1,123 active genes and found that they provide similar improvement of performance relative to random sampling (Supplementary Figure 20 A-B). The performance of OPEX is slightly better when running OPEX on the 1,123 active genes only, evidenced by the gap between two curves in Supplementary Figure 20A (p-value=0.02) and Supplementary Figure 20B (p-value=4×10^{-9}). However, the performance of OPEX on the active genes only is very close in two cases (Supplementary Figure 20C, p-value=0.02, and Supplementary Figure 20D, p-value=0.98). The relative improvement of MAE for OPEX compared to random sampling, is higher when OPEX making its prediction on all the genes (Supplementary Figure 19A versus Fig. 2B). This can be explained by the higher variance in the measurements among replicates when non-active genes are included (Supplementary Figure 21).

4.2.6 OPEX Comparison to Other OED Approaches

We compared the performance of OPEX with three alternative approaches, query by committee using different types of models, query by committee using bootstrapping, and D-optimal experimental design (see section 2.1 for more information in this file). The performance of each approach was evaluated

based on the average MAE of predictions for the expression of all the 4,391 genes (Supplementary Table 1 and Supplementary Figure 19). OPEX with entropy as utility function reached a better performance compared to query by committee using different types of models (QBC-Mixed-Models) when compared based on the maximum percentage of data points saved relative to random sampling (13 versus 14 iterations to reach MAE that random sampling achieves at iteration 27). With respect to the overall improvement of MAE relative to random sampling in all iterations, OPEX with entropy achieved 12.7% while QBC-Mixed-Models achieved 11.0% showing a slight advantage for OPEX with entropy (p-value $= 9 \times 10^{-9}$). OPEX with mutual information as the utility function performed similar to QBC-Mixed-Models. The query by committee using bootstrap QBC-Bootstrap and D-Optimal methods did not show a consistent advantage over random sampling. The prediction performance of the four types of models from query by committee was ranked in this order: Support Vector Regression, Gaussian process, linear regression and feed-forward neural network (Supplementary Figure 22).

4.3 Experimental Results

4.3.1 Exposure to Biocides and Cross-protection to Antibiotics

Our fitness measurements demonstrated that biocide treated *E. coli* cells, in majority of cases, exhibited cross-protection in antibiotics, excluding a couple of cases of cross-vulnerability. In 29 out of 40 treatment conditions, biocide treatment increased the fitness in antibiotic, while in 4 cases, treatment reduced the fitness. Cross-protection between biocides and antibiotics has been brought up to attention by researchers [21-23] and regulatory agencies [24-26] before. Biocides are regularly used as a sanitizer in hospitals, houses and food industries, and such study could help guide regulations to reduce the emergence of antimicrobial resistance.

Interestingly, pre-exposure to all biocides conferred protection against the antibiotic rifampicin (Fig. 3B), which was also the group that formed a cluster in the t-SNE/PCA analysis for transcriptomics of biocide/antibiotic pairs (Fig. 3A and Supplementary Figure 16). The highest fitness value was observed for the povidone iodine/kanamycin combination, and the lower for chlorophene/norfloxacin. These two extreme cases were selected for further investigations.

4.3.2 Three distinct clusters for all conditions

We examined cross-resistance of wild-type *E. coli* to each of the four antibiotics after pre-exposure to one of the ten biocides. Although there are 40 pairs of biocides and antibiotics, the gene expression (GE) profile was often dominated by only one factor (biocide or antibiotic). The dominating factor can be explained using three rules as evident by the three clusters in Fig. 3A. First, the alcohol biocides (Ethanol, Isopropanol and Chlorhexidine) had a dominating effect on GE profile regardless of the antibiotic they are paired with. Second, apart from the alcohols, in the majority of cases, rifampicin had a dominating effect on GE profile regardless of the biocide that it was paired. Third, the choice of biocide determined GE profile except when Rifampicin was used. This is evident by the proximity of points related to each biocide Benzalkonium chloride, Chlorhexidine, Chlorophene, Ethanol, Glutaraldehyde, H_2O_2, Isopropanol and Peracetic acid on the t-SNE plot (Fig 3A). The same clusters were also detected by the PCA plot (Supplementary Figure 17). The kanamycin/ peracetic acid pair is particularly interesting since it did not follow the general pattern. We further asked whether these clustering patterns can be explained based on Biological processes (BPs), especially w.r.t. to clustering of kanamycin/ peracetic acid pair. Enrichment of BPs was performed using the GSEA, and subsequently, hierarchical clustering was performed, which demonstrated that in the majority of cases, clustering on GE profiles and BPs follow a similar trend and kanamycin/ peracetic acid pair remains with the rifampicin cluster (Supplementary Figure 23).

Supplementary Tables

Supplementary Table 1. Mode of actions of biocides and antibiotics

S.No.	Biocide	Key mode of action	Reference
1	Ethanol	Lipid and protein denaturation	[27]
2	Isopropanol	Lipid and protein denaturation	[28]
3	Chlorophene	Lipid and protein denaturation	[29]
4	Glutaraldehyde	Alkylation of hydroxyl, carbonyl, and amino group, affecting protein, DNA, cell membrane	[30]
5	Peracetic acid	Oxidation	[31]
6	H2O2	Oxidation	[31]
7	Sodium hypochlorite	Oxidation	[32]
8	Chlorhexidin	Positively charged. Binds with negatively charged cell and membrane and damages them, causes cytoplasm solidification	[33]
9	Benzalkonium chloride	Cationic surfactant. Binds with negatively charged cell membrane and damages it. Denatures protein.	[34]
10	Povidone Iodine	Iodination of membrane lipids, oxidation of cytoplasmic and membrane proteins	[35]
**S.No.**	**Antibiotic**	**Key mode of action**	
1	Ampicillin	Cell wall synthesis inhibitor, inhibits transpeptidase	[36]
2	Rifampicin	Transcription inhibitor, binds and inhibits RNA pol	[36]
3	Kanamycin	Translation inhibitor, binds and inhibits 30S rRNA	[37]
4	Norfloxacin	Cell division inhibitor, binds and inhibits DNA gyrase and topoisomerase iv	[38]

Supplementary Table 2. OPEX Compared to Other OED Approaches. The predictive performance was evaluated using the maximum percentage of data saved using the given OED method compared to random sampling. The QBC-Bootstrap and D-Optimal methods performed better or worse than random depending on the iteration as showcased by their zero performance. GP: gaussian process; LR: linear regression; SVR: support vector regression; FNN: feedforward neural network. All 4,391 genes were used in for evaluations here.

OED Method	Utility Function	Predictive Model	Performance
1. OPEX	Entropy	GP	48% (13 vs 27)
2. OPEX	Mutual Information	GP	44% (14 vs 27)
3. QBC-Mixed-Models	Variance Among Committee	GP	44% (14 vs 27)
4. QBC-Bootstrap	Variance Among Committee	GP	0% (not better)
5. D-Optimal	Determinant of Fisher Information	LR	0% (not better)

Supplementary Figures

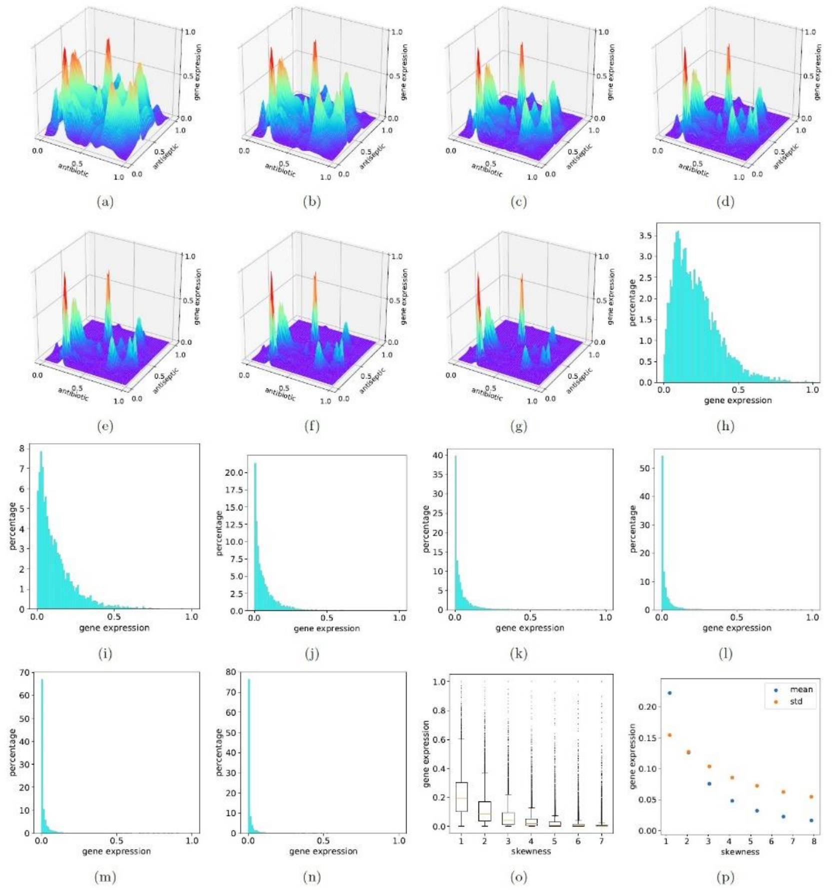

Supplementary Figure 1: Visualization of the seven synthetic datasets that have similar topology but different skewness in the distribution of the output (gene expression dimension). Panel a to g corresponds to dataset 1 to 7 and the skewness is 1.17, 2.07, 3.05, 4.13, 5.29, 6.55 and 7.86, respectively. Panel h to n is the histogram of the output for the seven datasets (every dataset consists of 6400 datapoints). Panel o shows the distribution of the output for the datasets. Panel p shows the mean and standard deviation of the output versus the skewness of each dataset. In panel k, each box represents an interquartile range which consists of data points between the 25th and 75th percentiles. The whiskers extend to the maximum and minimum values but no further than 1.5 times of the interquartile range for a given whisker. The horizontal line within each box represents the median.

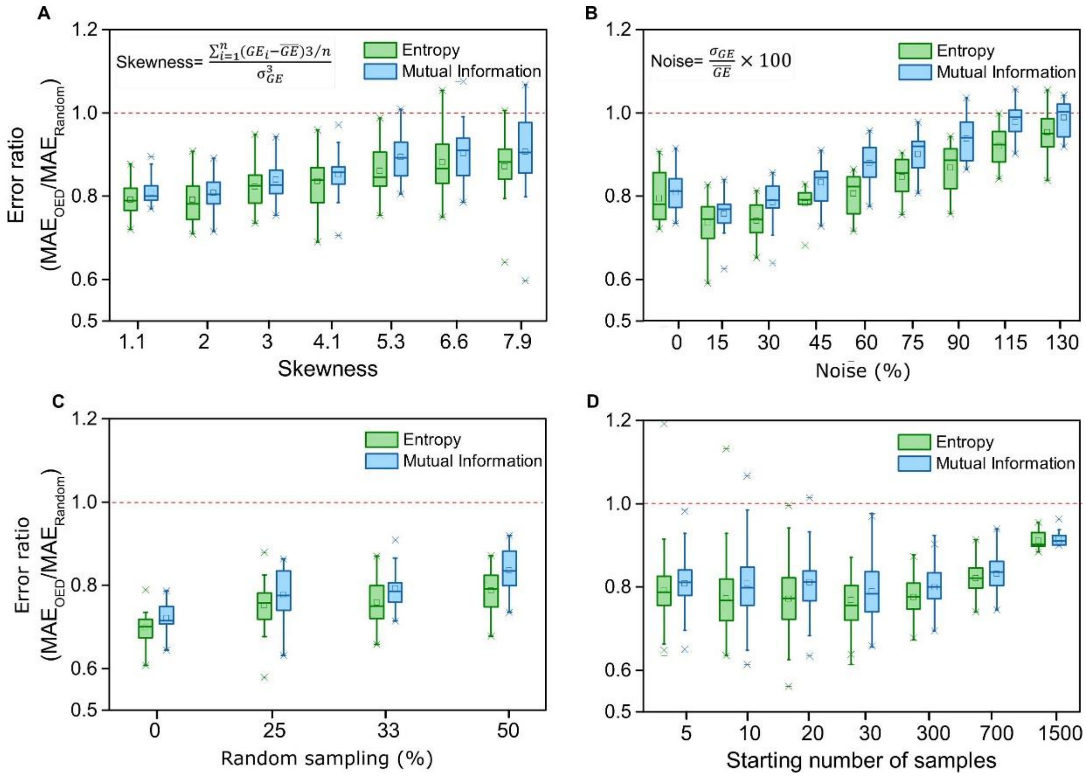

Supplementary Figure 2: A. The performance of OED methods after sampling 150 additional datapoints versus the skewness of the synthetic dataset. The performance of an OED method was evaluated by the difference in MAE between an OED method and random sampling normalized by the MAE by the OED method. Other variables in the setting: noise level = 20%, start size = 300, exploration frequency = 1/3, batch size = 3. **B-D**. The performance of the OED methods versus other parameters as the x axis of each panel describes when tested on the dataset whose skewness is 1.1 in panel A. Parameters other than the parameter investigated in a panel and exploration frequency are the same as that in panel A. The number of datapoints for each box in the boxplot is 50. Each box represents an interquartile range which consists of data points between the 25th and 75th percentiles. The whiskers extend to the maximum and minimum values but no further than 1.5 times of the interquartile range for a given whisker. The horizontal line within each box represents the median.

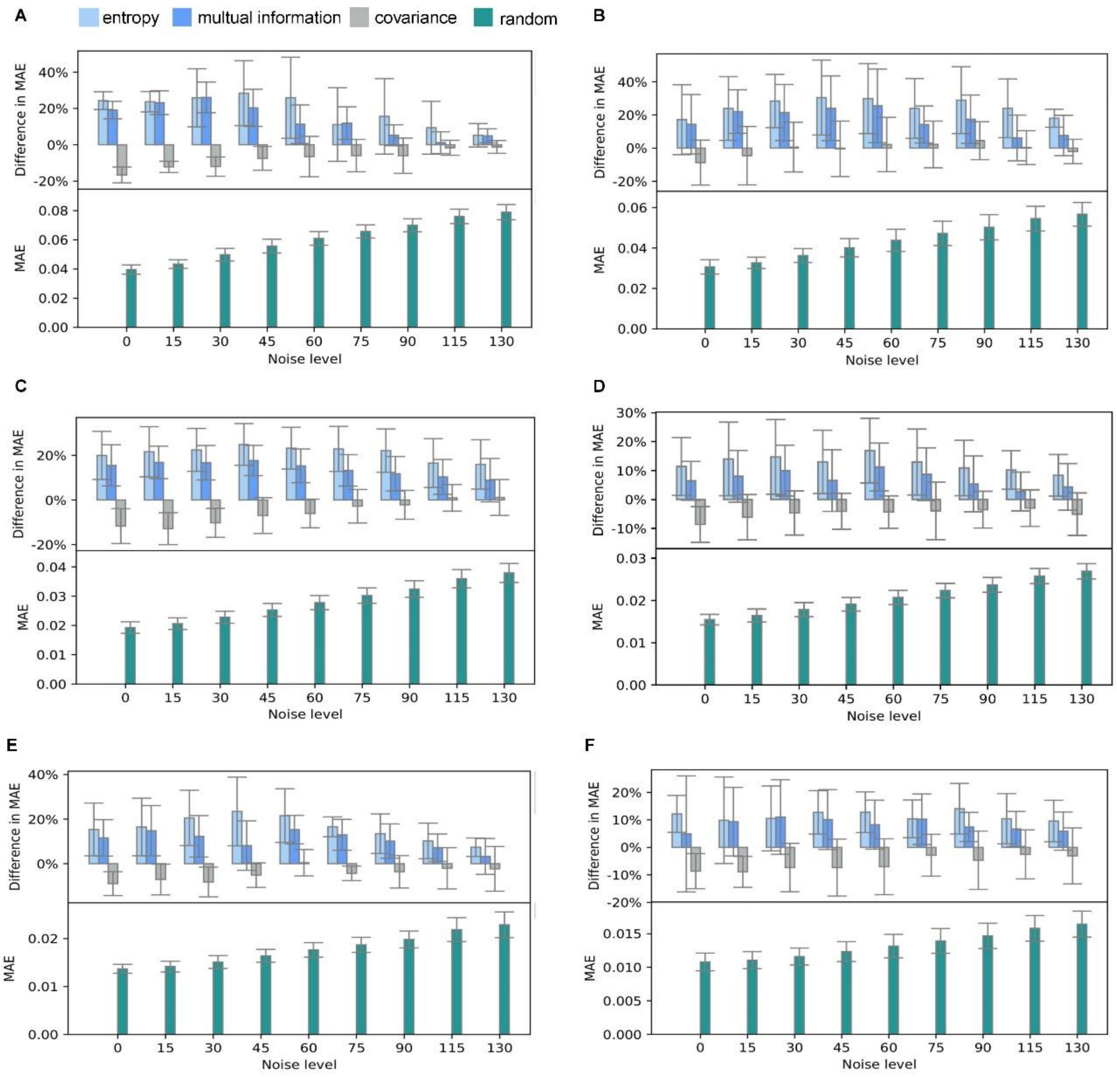

Supplementary Figure 3: The effect of noise on the performance of the OED methods compared to random sampling on synthetic datasets 2-7 (A- F), whose skewness are 2.07, 3.05, 4.13, 5.29, 6.55 and 7.06, respectively. The setting for other hyper-parameters are as follows: starting size=300, exploration frequency=1/6, batch size=3, the number of iterations=50. The error bar denotes standard deviation (number of datapoints=50). The bar represents the mean of 50 runs.

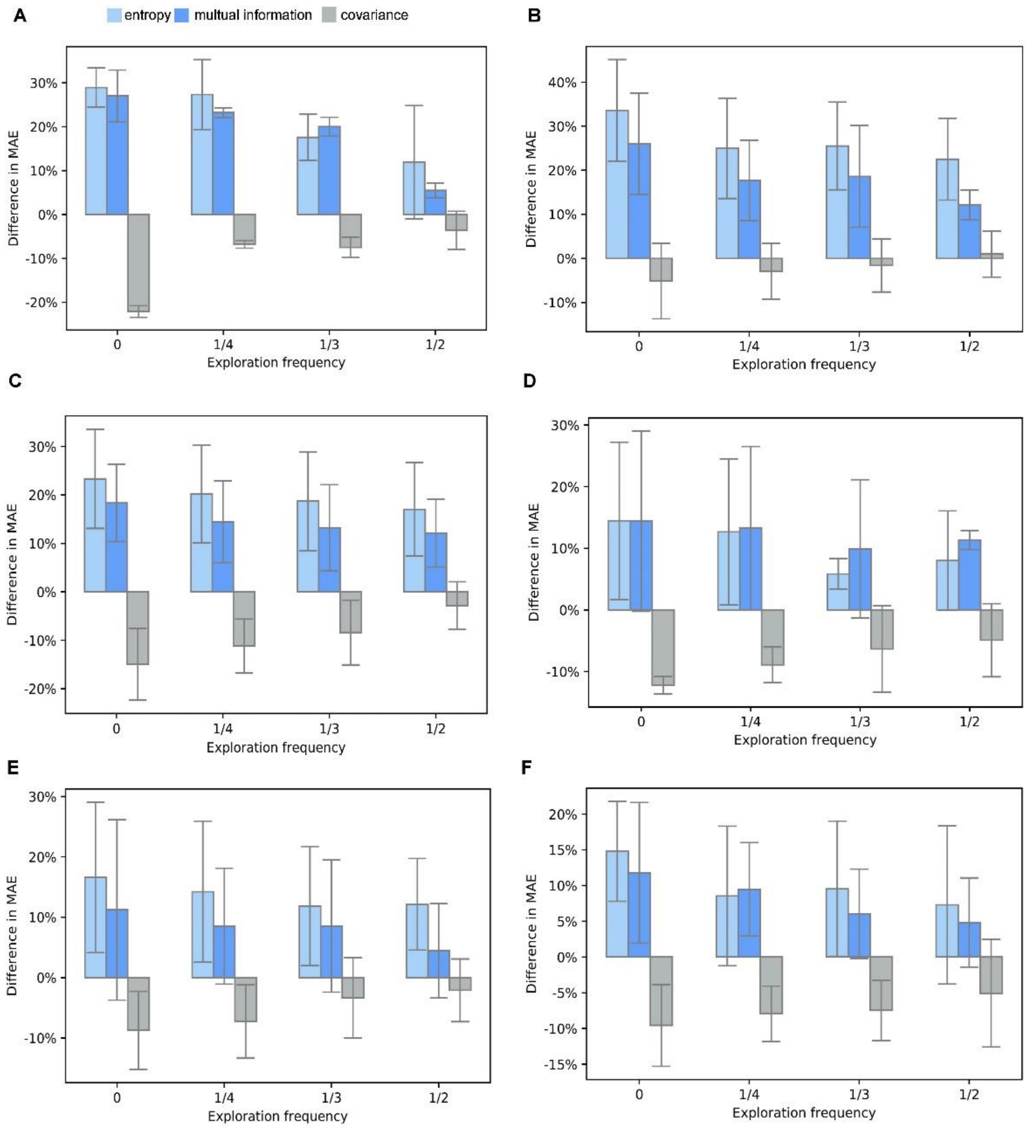

Supplementary Figure 4 A-F: The effect of exploration frequency on the performance of the OED methods compared to random sampling on synthetic datasets 2-7, whose skewness are 2.07, 3.05, 4.13, 5.29, 6.55 and 7.06, respectively. The y-axis is the MAE of random sampling minus the MAE of OPEX divided by the MAE of random sampling. A positive value means OPEX is more effective. The setting for other hyper-parameters are as follows: starting size=300, noise level=20, batch size=3, the number of iterations=50. The error bar denotes standard deviation (number of datapoints=50). The bar represents the mean of 50 runs.

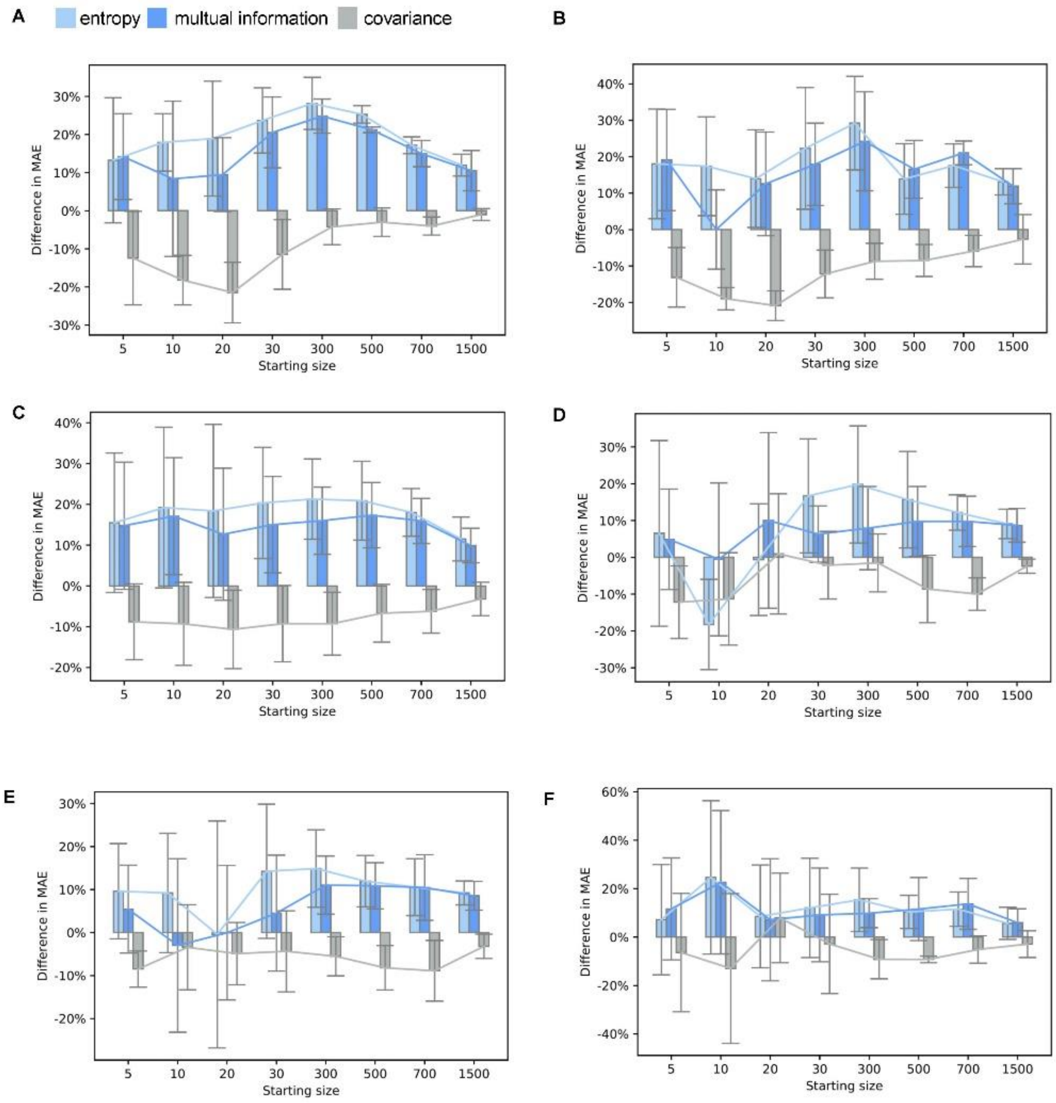

Supplementary Figure 5: The effect of starting size on the performance of the OED methods compared to random sampling on datasets 2-7 (**A-F**), whose skewness are 2.07, 3.05, 4.13, 5.29, 6.55 and 7.06, respectively. The setting for other hyper-parameters are as follows: noise level=20, batch size=3, minimum distance=0.2, exploration frequency=1/6, the number of iterations=50. The error bar denotes standard deviation (number of datapoints=50). The bar represents the mean of 50 runs.

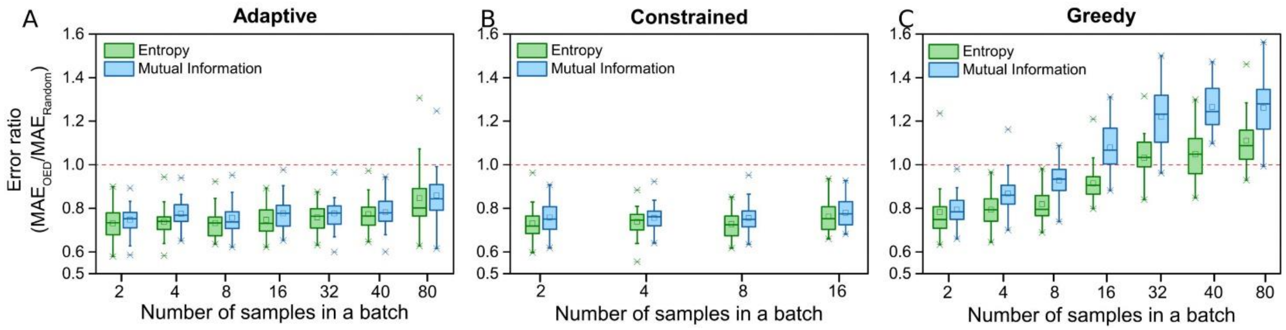

Supplementary Figure 6: Performance of adaptive sampling, constrained sampling and greedy sampling on the data whose skewness is 1.1. The setting for other hyper-parameters are as follows: starting size=300, noise level=20, exploration frequency=1/6, the number of total additional datapoints sampled=160. For Panel B, the minimum distance between datapoints in a batch is 0.2. As the batch size, k, goes beyond 16, we cannot select k points with pairwise distances greater than 0.2 hence the x-axis is from 2 to 16 in Panel B. The number of datapoints for each box in the boxplot is 50. Each box represents an interquartile range which consists of data points between the 25th and 75th percentiles. The whiskers extend to the maximum and minimum values but no further than 1.5 times of the interquartile range for a given whisker. The horizontal line within each box represents the median.

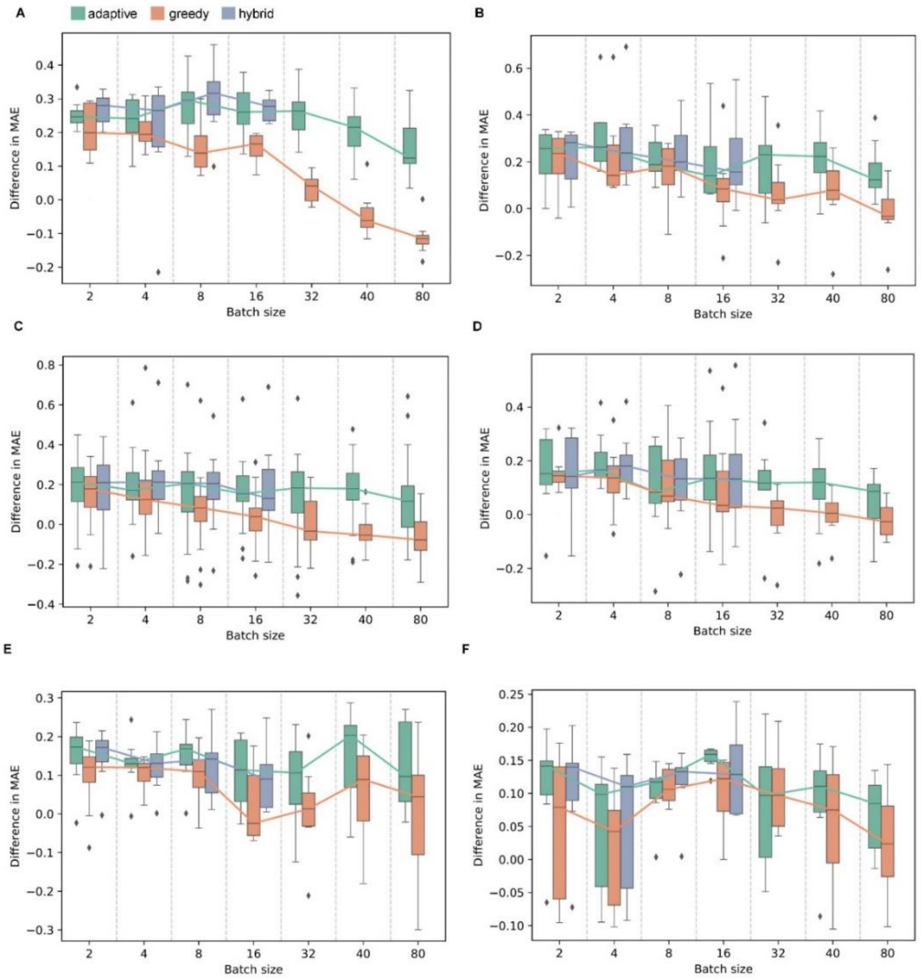

Supplementary Figure 7 A-F: the effect of batch size on the performance of the OPEX compared to random sampling on datasets 2-7, whose skewness are 2.07, 3.05, 4.13, 5.29, 6.55 and 7.06, respectively. The setting for other hyper-parameters are as follows: starting size=300, noise level=20, minimum distance=0.2, exploration frequency=1/6, the number of total additional datapoints sampled=160. The utility function used by OPEX was entropy. adaptive, greedy, and hybrid represents adaptive sampling, greedy sampling and hybrid sampling, respectively. The number of datapoints for each box in the boxplot is 50. Each box represents an interquartile range which consists of data points between the 25th and 75th percentiles. The whiskers extend to the maximum and minimum values but no further than 1.5 times of the interquartile range for a given whisker. The horizontal line within each box represents the median.

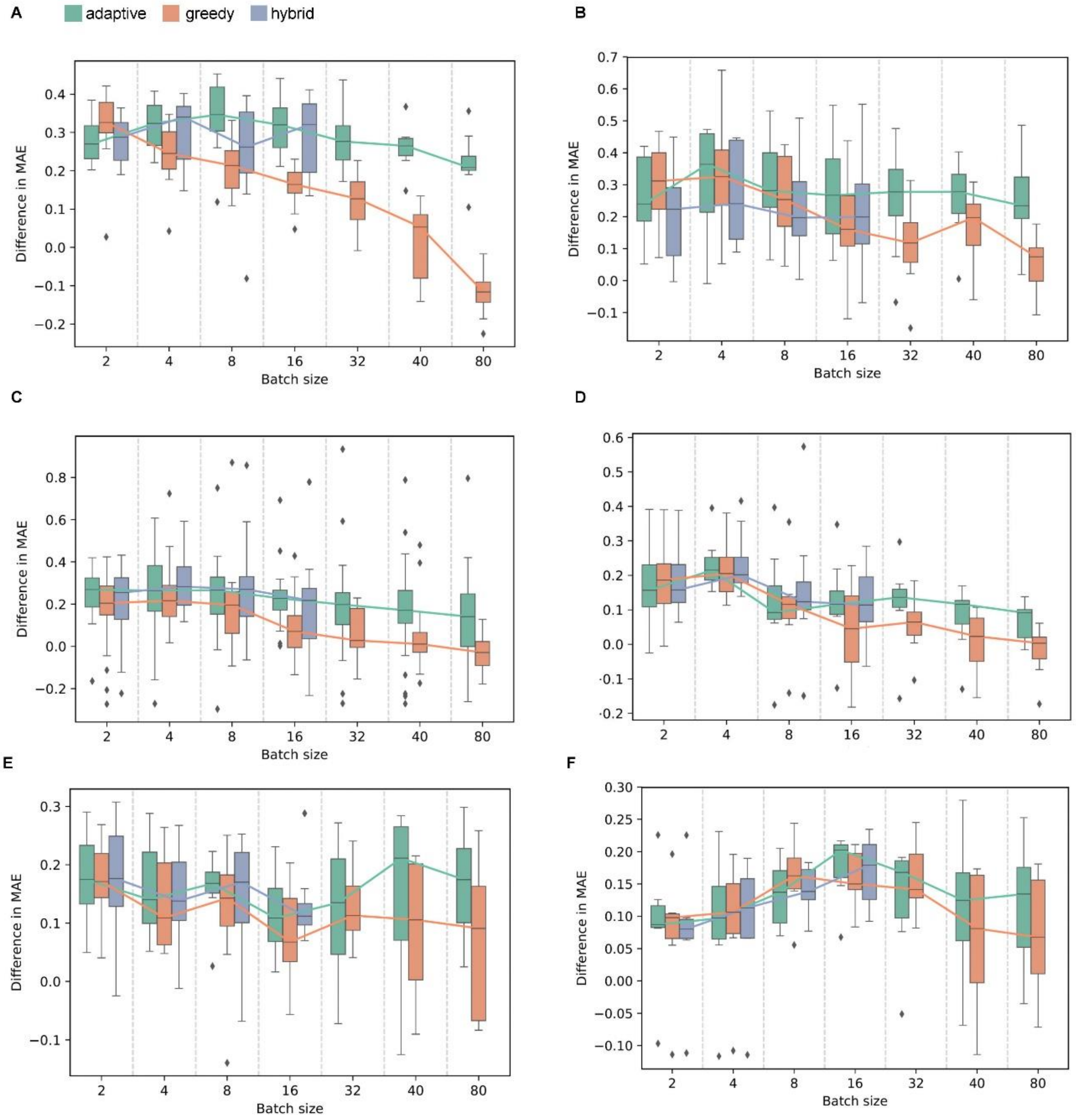

Supplementary Figure 8: It is the same as Supplementary Figure 8 except that the utility function is mutual information used by OPEX.

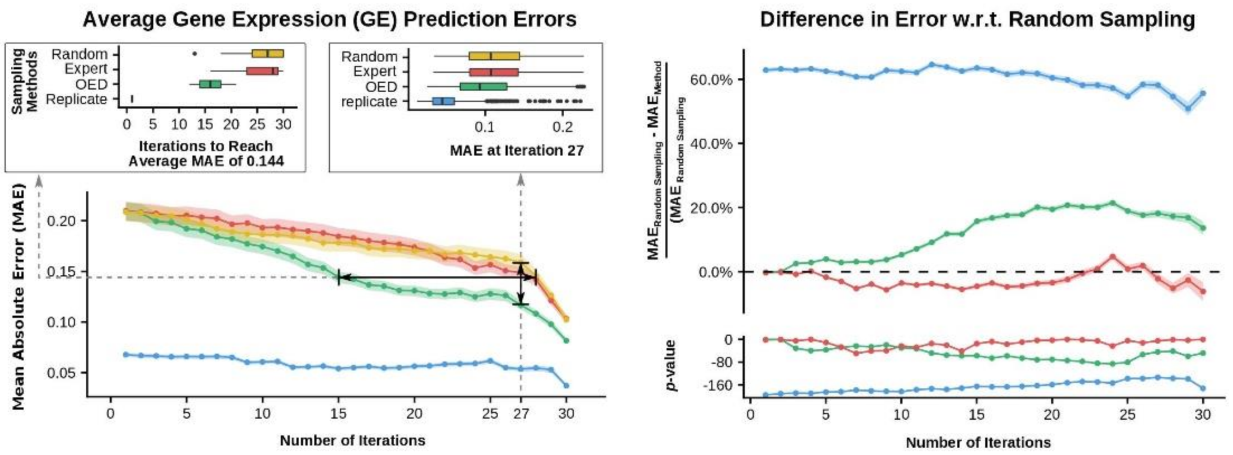

Supplementary Figure 9: The performance of OPEX on real dataset with 50% exploration frequency. It is the same as Figure 2 B & C, except that the utility function used here is entropy.

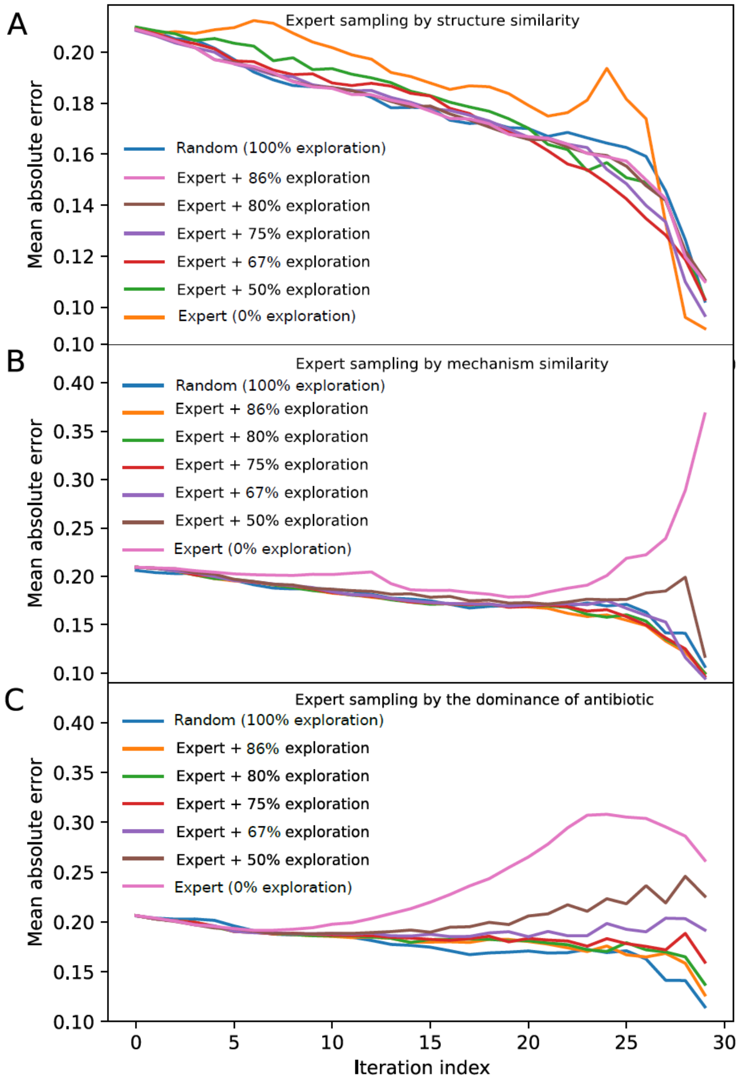

Supplementary Figure 10: The performance of three expert sampling approaches. We used expert sampling to sample one datapoint every k iterations and used random sampling otherwise to introduce exploration. The percentage of exploration in the legends is equal to k/(k+1). E.g. 67%=2/(2+1). (**A**) Structure similarity means that the culture condition that was most dissimilar to all the observed conditions was selected in each iteration. The similarity between two culture conditions is quantified by the structure similarity between the biocides used in the two conditions and the antibiotics used. (**B**) Mechanism similarity means the mechanism of each antibiotic and biocide was considered when selecting the most dissimilar culture condition. (**C**) Dominance of antibiotic means the more dominant an antibiotic is, the later a culture condition that has that antibiotic condition was selected.

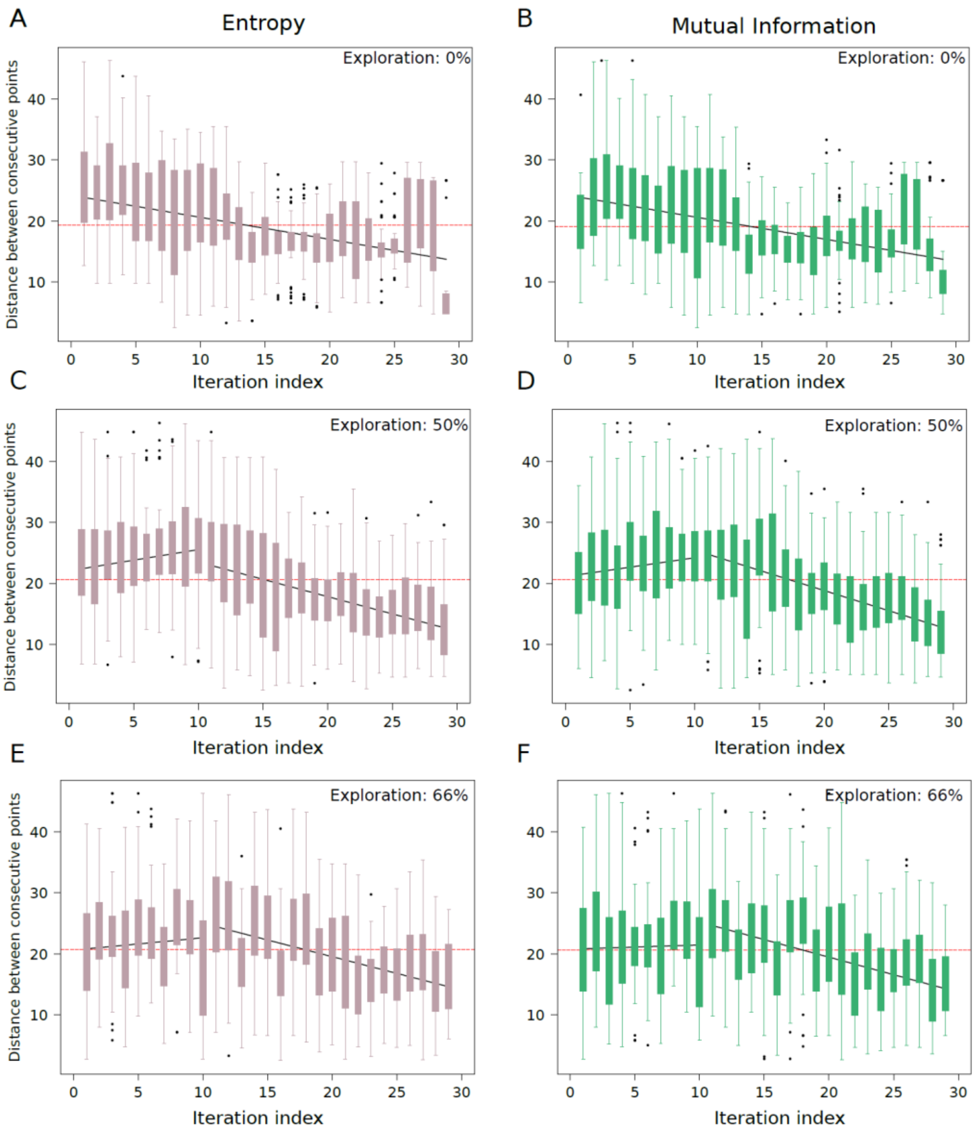

Supplementary Figure 11: The distance between the GE profiles of selected datapoints in every two adjacent iterations by OPEX with various percentages of exploration. The number on the top left corner in each panel represents the percentage of exploration used by OPEX. We sampled one datapoint every k iterations based on the entropy or mutual information and used random sampling otherwise to introduce exploration. The percentage of exploration in the legend is equal to k/(k+1). E.g. 66%=2/(2+1). 0% means always selecting a datapoint based on entropy or mutual information. The number of datapoints for each box in the boxplot is 50. Each box represents an interquartile range which consists of data points between the 25th and 75th percentiles. The whiskers extend to the maximum and minimum values but no further than 1.5 times of the interquartile range for a given whisker. The horizontal line within each box represents the median.

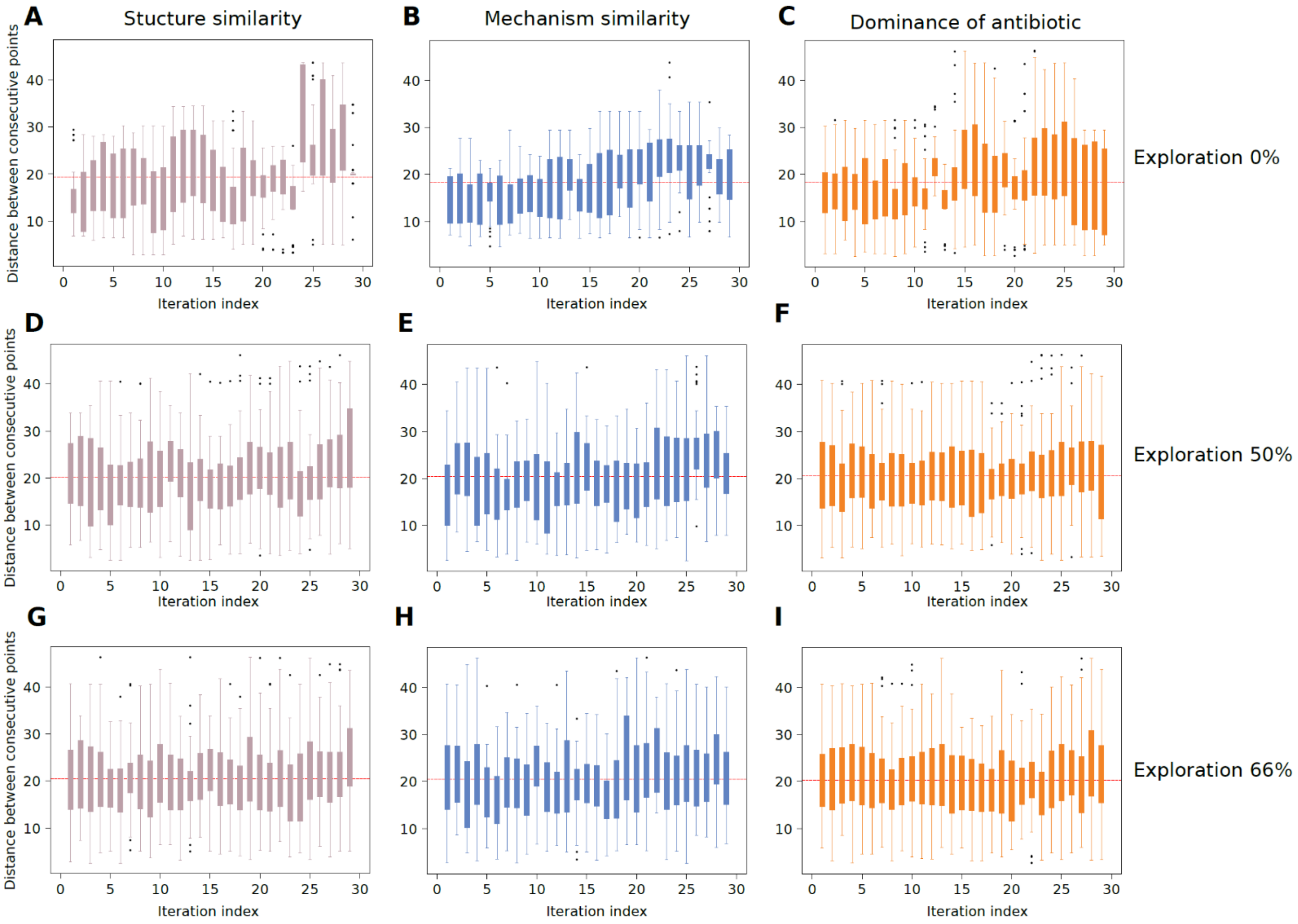

Supplementary Figure 12: The distance between the select datapoints in every two adjacent iterations by expert sampling with various percentage of exploration. The number on the right in each row of panels represents the percentage of exploration used. We sampled one datapoint every k iterations based on expert sampling and used random sampling otherwise to introduce exploration. The percentage of exploration in the legend is equal to k/(k+1). E.g. 66%=2/(2+1). The number of datapoints for each box in the boxplot is 50. The definition of box plot is defined the same way as in Supplementary Figure 11.

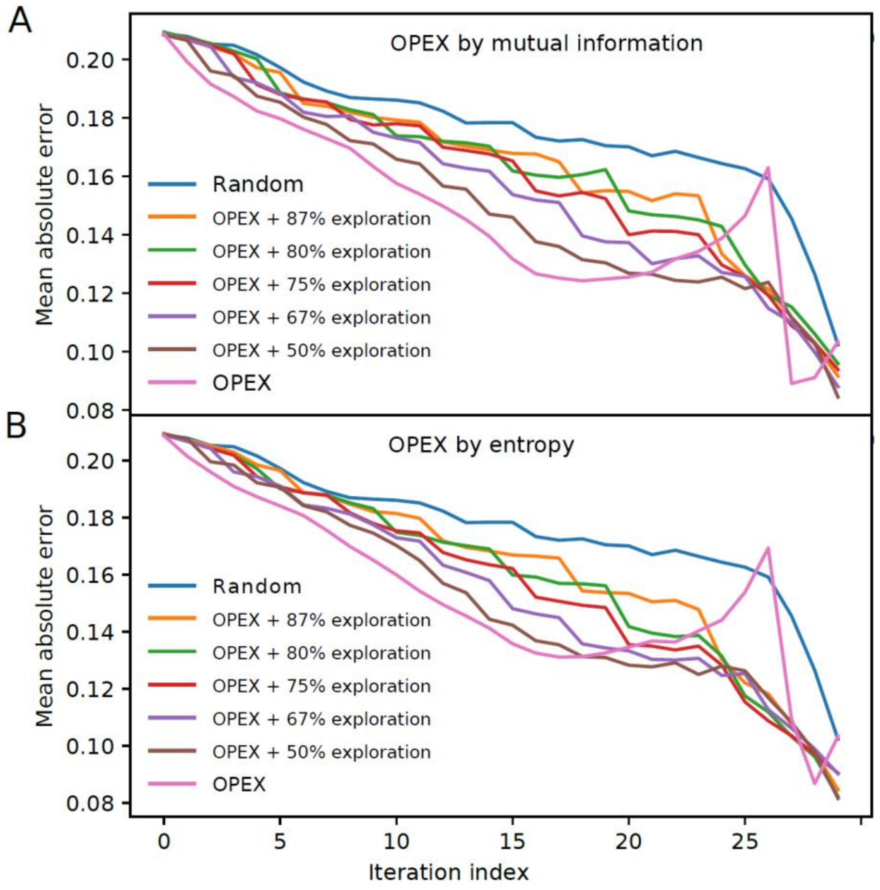

Supplementary Figure 13: The performance of OPEX which used mutual information **(A)** or entropy **(B)** as the utility function. The effect of the tradeoff between exploration and exploitation on the performance of OPEX was visualized. We sampled one datapoint every k iterations based on the entropy or mutual information and used random sampling otherwise to introduce exploration. The percentage of exploration in the legends is equal to k/(k+1). E.g. 67%=2/(2+1).

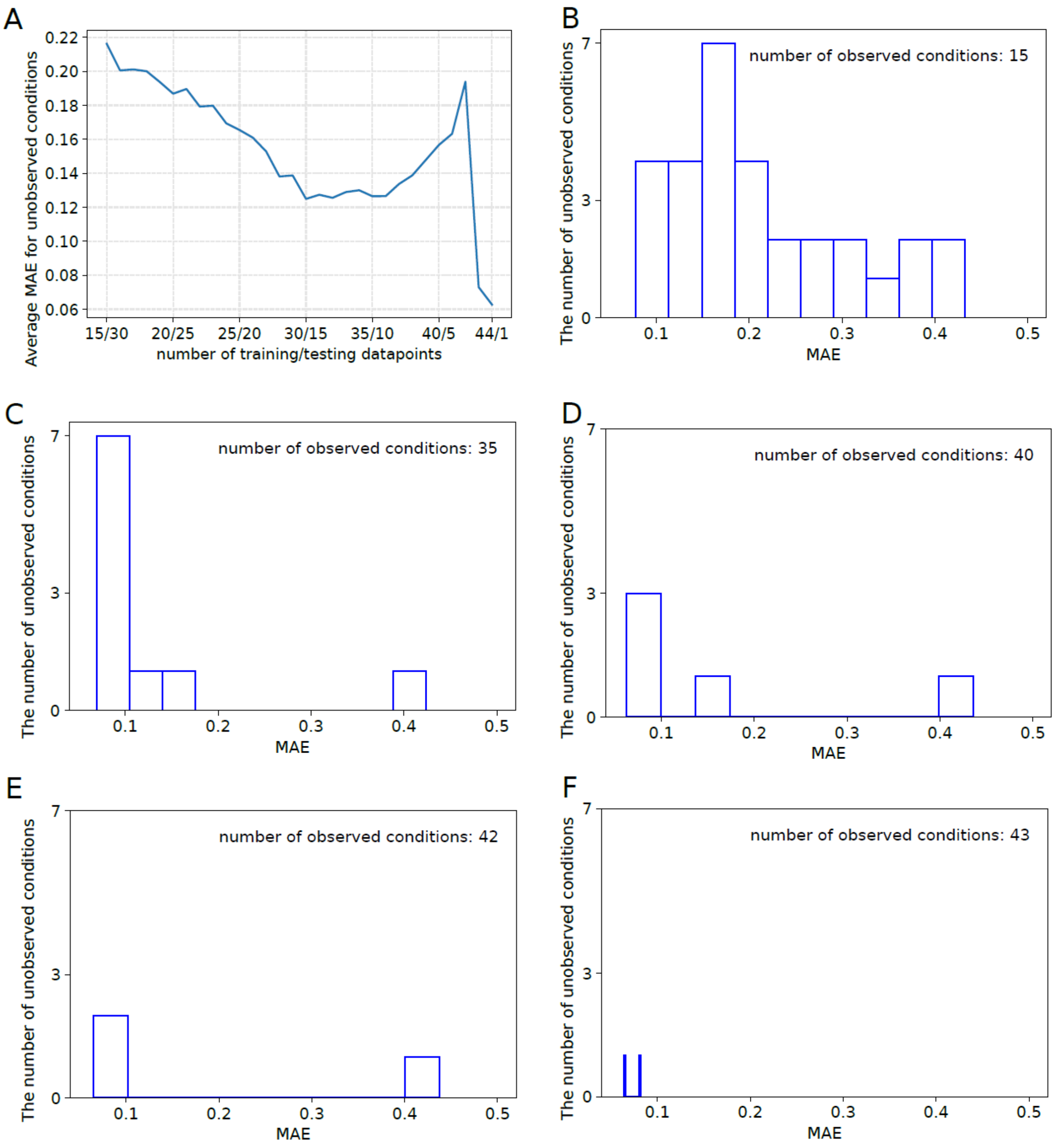

Supplementary Figure 14: All the panels in this Figure were associated with one OPEX run randomly picked (summary of all OPEX runs is in Supplementary Figure 15). **A.** The learning curve of OPEX which used 0% exploration. **B-F.** The histogram of the average MAE of predicted gene expression for all the remaining conditions at different iterations.

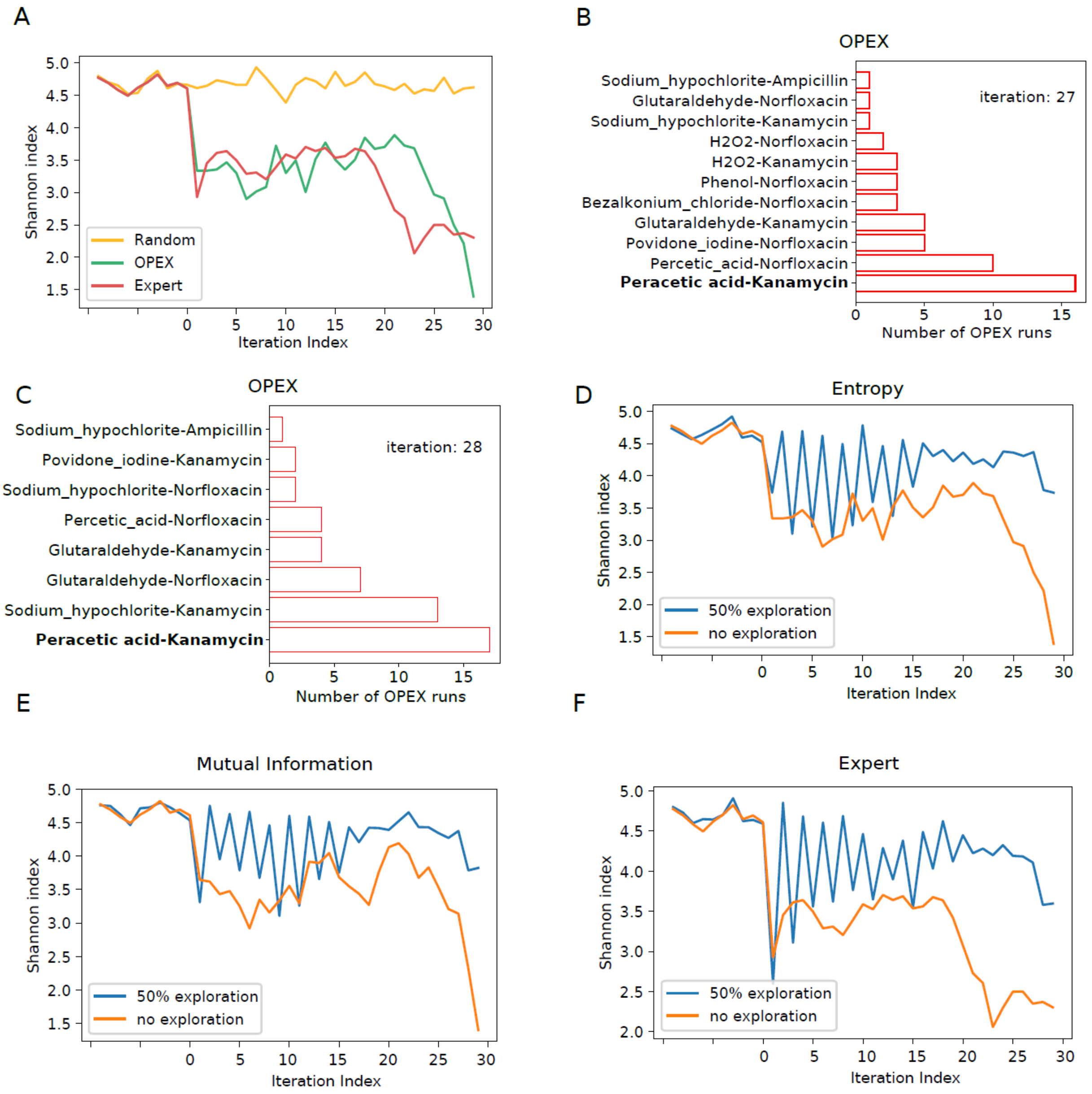

Supplementary Figure 15: All the panels were based on 50 simulations. **A.** The Shannon index represents the diversity amongst conditions selected at a specific iteration in 50 OPEX runs. The curves before the iteration 0 are about the 10 conditions randomly selected as part of the starting training set. The other 5 conditions in the starting training set are the control and antibiotic only conditions. The percentage of explration is defined the same way as in Supplementary Figure 13. **B&C**. The histogram of the conditions selected as the 27th/28th iteration **D-F**. The Shannon index of the conditions selected at each iteration by OPEX and expert sampling. The curves before iteration 0 is defined the same as in panel A.

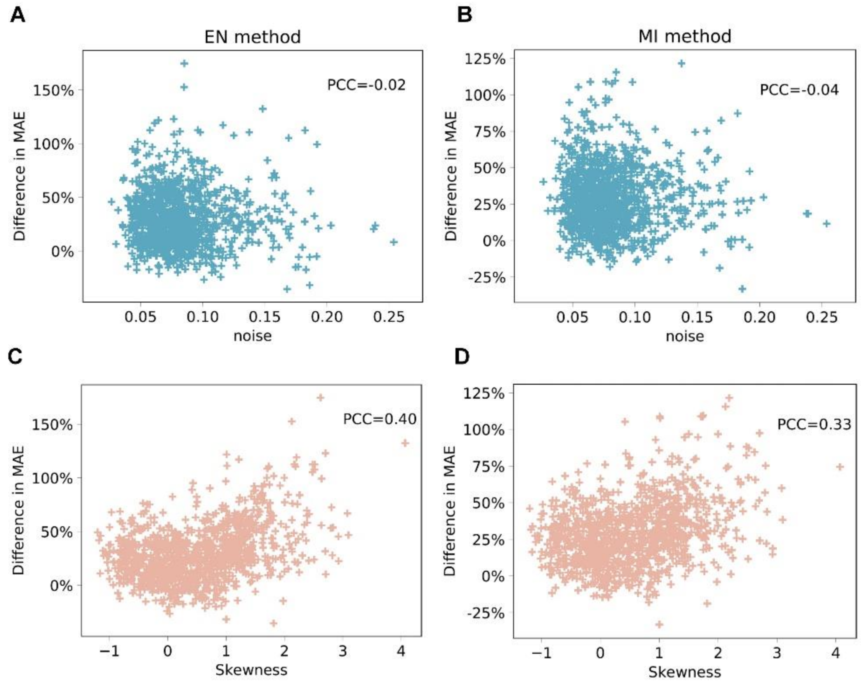

Supplementary Figure 16: Each point represents a single gene in all four plots. The vertical axis represents the relative difference between EN/MI and Random sampling with respect to the average MAE of GE predictions for unobserved conditions at iteration 15. Higher difference in MAE is associated with better performance of OPEX when compared to random sampling. For **A&B,** the horizontal axis represents the noise of GE amongst replicates. For **C&D** the horizontal axis represents the skewness related to the distribution of GE values of a given gene in all conditions.

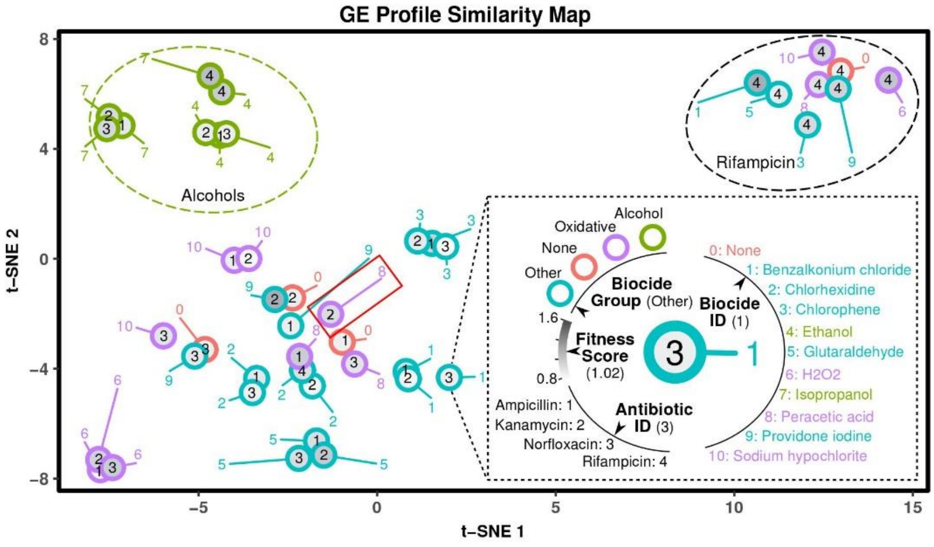

Supplementary Figure 17: This Figure is same as the Fig. 3A except that the GE profile of the peracetic acid and kanamycin condition was predicted by GP models trained on all other conditions. That condition is highlighted by red box which was originally in the cluster named rifampicin in Fig. 3A.

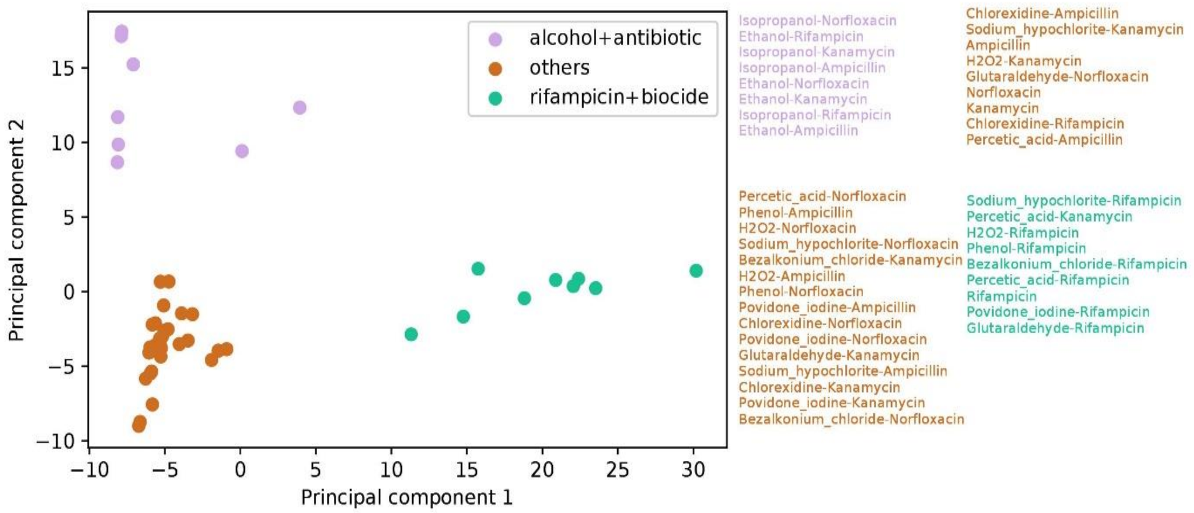

Supplementary Figure 18: A visualization of the culture conditions in the first two principal components. The input is gene expression profiles when running PCA. The membership of each condition was color coded.

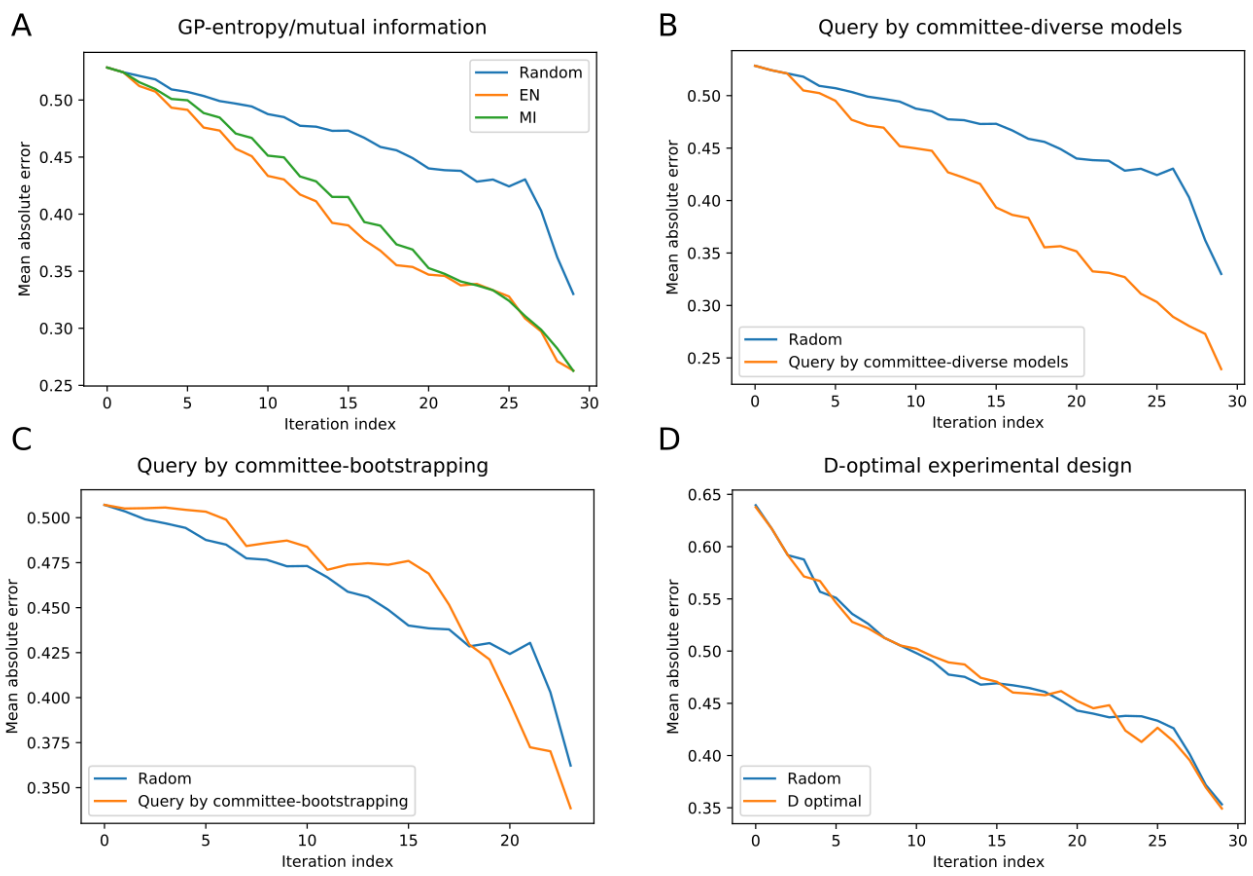

Supplementary Figure 19: The prediction performance of different optimal experimental design approaches. **A.** OPEX. **B.** At each iteration four types of model, feed forward neural network, linear regression, Support Vector Regression and linear regression, are trained. The one which has the largest variance among the prediction by the four models is selected among all the unobserved culture conditions. When generating the learning curve, the GP model is used to get a fair comparison with the result in A. For the prediction performance of each type of model, see Supplementary Figure 22. **C:** When building a committee, 4 different datasets are created from the training set at each iteration and four GP models are trained. To get a more diversified bootstrapping dataset, the size of the starting training set is 20, whereas it is 15 in other panels. Finally, the most uncertain one among the committee is selected. **D.** A linear model is trained and the next culture condition is selected through D-optimal experimental design. Note that all the genes were used when generating the learning curves.

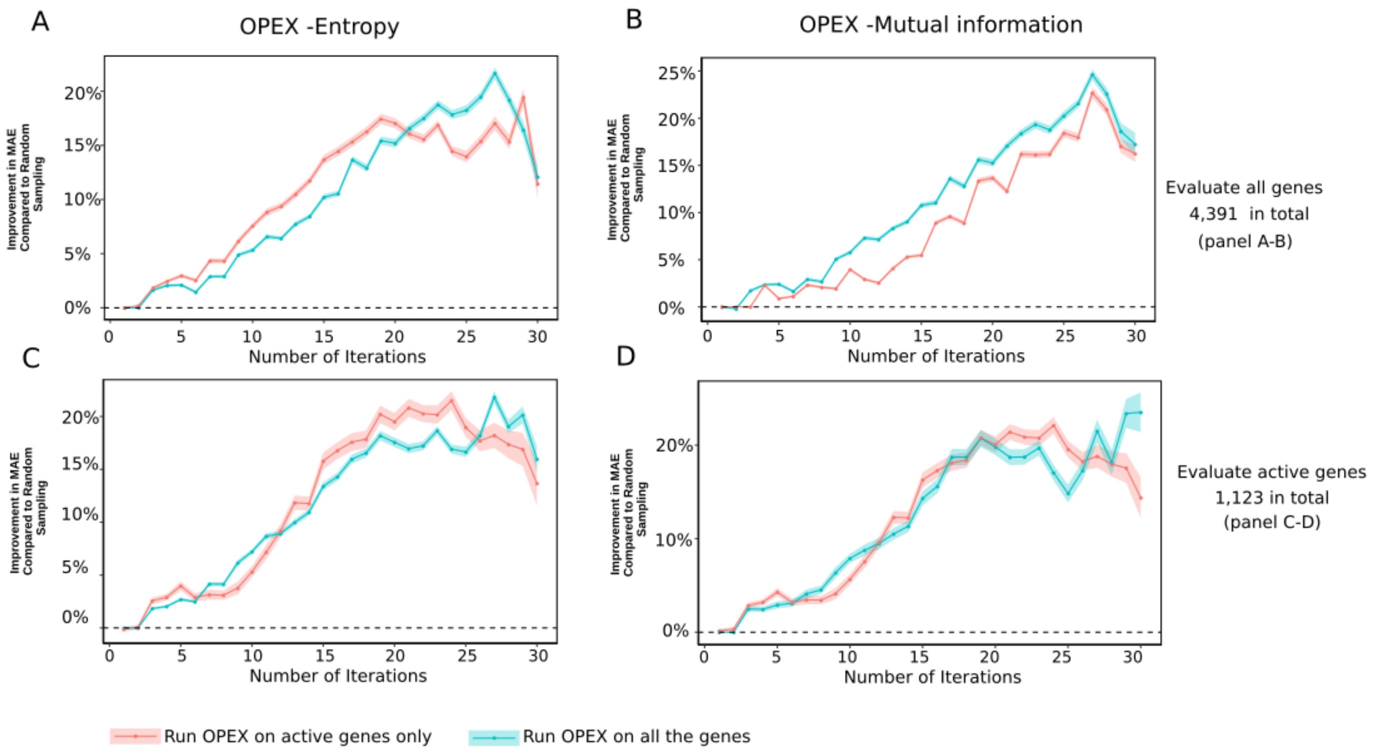

Supplementary Figure 20: The performance of OPEX variants, where all 4,391 genes (cyan) or only the high-CPM 1,123 genes (red) are taken into account when selecting the batch at each iteration. All plots show the relative difference in MAE between OPEX and random sampling. (**A-B**). MAE measured all genes, (**C-D**) MAE measured only active genes. The shadow envelope around each curve represents 95% confidence interval.

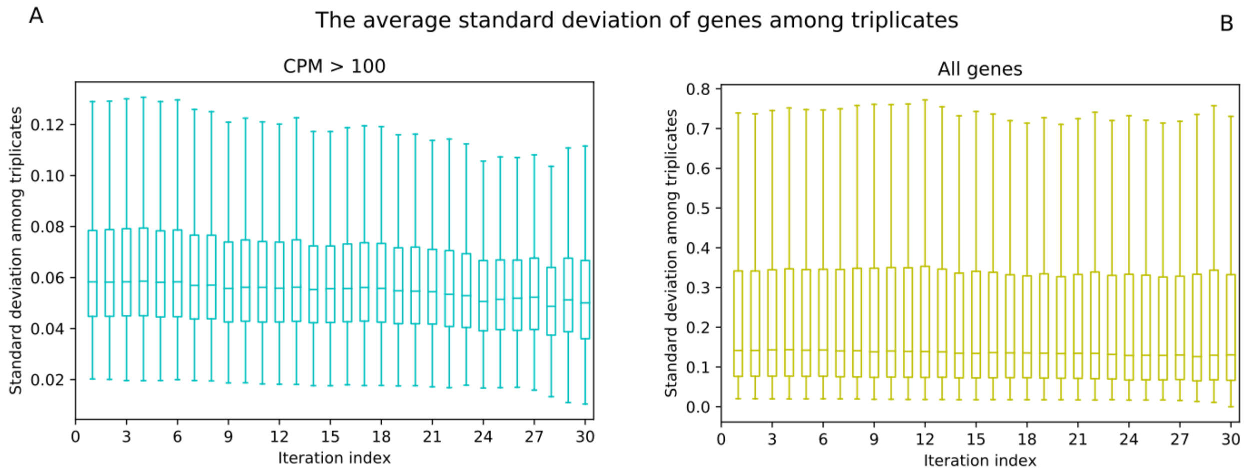

Supplementary Figure 21: The average variance in the measured gene expression among the three replicates for each culture condition. Only the genes that have a count per million larger than 100 in half of all the datapoints were used (A) (1,123 genes in total). All the 4,391 genes are used (B). The number of datapoints for each box in the boxplot is 50. Each box represents an interquartile range which consists of data points between the 25th and 75th percentiles. The whiskers extend to the maximum and minimum values but no further than 1.5 times of the interquartile range for a given whisker. The horizontal line within each box represents the median.

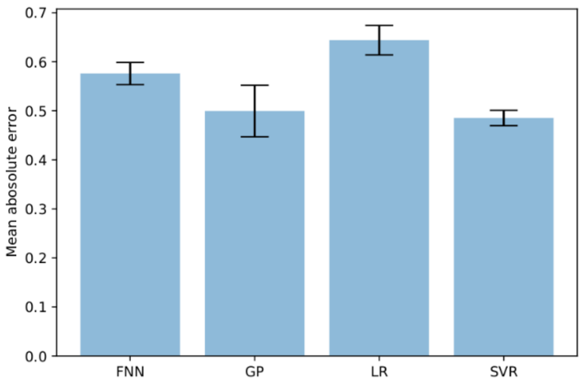

Supplementary Figure 22: The prediction performance of four types of machine learning models. FNN: feed forward neural network; GP: Gaussian process; LR: linear regression; SVR: Support Vector Regression. When evaluating the performance of a type of model, a model was trained using 15 randomly selected conditions and the mean absolute error in the predicted gene expression profile on the remaining culture conations was recorded. An error bar represents standard deviation of 50 runs

Supplementary References

1. Rasmussen, C. E. Gaussian processes in machine learning. In *Summer School on Machine Learning*, 63–71 (Springer, 2003).
2. Guestrin, C., Krause, A. & Singh, A. P. Near-optimal sensor placements in gaussian processes. In *Proceedings of the 22nd international conference on Machine learning*, 265–272 (2005).
3. Bajusz, D., Rácz, A. & Héberger, K. Why is tanimoto index an appropriate choice for fingerprint-based similarity calculations? *J. cheminformatics* **7**, 20 (2015).
4. Campbell, E. A. *et al.* Structural mechanism for rifampicin inhibition of bacterial rna polymerase. *Cell* **104**, 901–912 (2001).
5. von Ahsen, U., Davies, J. & Schroeder, R. Antibiotic inhibition of group i ribozyme function. *Nat.* **353**, 368–370 (1991).
6. Benbrook, D. M. & Miller, R. V. Effects of norfloxacin on dna metabolism in pseudomonas aeruginosa. *Antimicrob. agents chemotherapy* **29**, 1–6 (1986).
7. Kaldalu, N., Mei, R. & Lewis, K. Killing by ampicillin and ofloxacin induces overlapping changes in escherichia coli transcription profile. *Antimicrob. agents chemotherapy* **48**, 890–896 (2004).
8. Robinson, M. D., McCarthy, D. J. & Smyth, G. K. edger: a bioconductor package for differential expression analysis of digital gene expression data. *Bioinforma.* **26**, 139–140 (2010).
9. Cohn, D. A. Neural network exploration using optimal experiment design. In *Advances in neural information processing systems*, 679–686 (1994).
10. Pearson, K. On lines and planes of closest fit to systems of points in space, 1901. *DOI* **10**, 45 (1975).
11. Maaten, L. v. d. & Hinton, G. Visualizing data using t-sne. *J. machine learning research* **9**, 2579–2605 (2008).
12. McDonnell, G. & Russell, A. D. Antiseptics and disinfectants: activity, action, and resistance. *Clin. microbiology reviews* **12**, 147–179 (1999).
13. Baba, T. *et al.* Construction of escherichia coli k-12 in-frame, single-gene knockout mutants: the keio collection. *Mol. systems biology* **2** (2006).
14. Datsenko, K. A. & Wanner, B. L. One-step inactivation of chromosomal genes in escherichia coli k-12 using pcr products. *Proc. Natl. Acad. Sci.* **97**, 6640–6645 (2000).
15. Subramanian, A. *et al.* Gene set enrichment analysis: a knowledge-based approach for interpreting genome-wide expression profiles. *Proc. Natl. Acad. Sci.* **102**, 15545–15550 (2005).
16. Huang, D. W., Sherman, B. T. & Lempicki, R. A. Bioinformatics enrichment tools: paths toward the comprehensive functional analysis of large gene lists. *Nucleic acids research* **37**, 1–13 (2009).
17. Raffa, R. G. & Raivio, T. L. A third envelope stress signal transduction pathway in escherichia coli. *Mol. microbiology* **45**, 1599–1611 (2002).
18. Braoudaki, M. & Hilton, A. Adaptive resistance to biocides in salmonella enterica and escherichia coli o157 and cross-resistance to antimicrobial agents. *J. clinical microbiology* **42**, 73–78 (2004).
19. Langsrud, S., Sundheim, G. & Holck, A. Cross-resistance to antibiotics of escherichia coli adapted to benzalkonium chloride or exposed to stress-inducers. *J. applied microbiology* **96**, 201–208 (2004).

20. Tattawasart, U., Hann, A., Maillard, J.-Y., Furr, J. & Russell, A. Cytological changes in chlorhexidine-resistant isolates of pseudomonas stutzeri. *J. Antimicrob. Chemother.* **45**, 145–152 (2000).

21. Pereira, B. M. P. & Tagkopoulos, I. Benzalkonium chlorides: Uses, regulatory status, and microbial resistance. *Appl. environmental microbiology* **85**, e00377–19 (2019).

22. Yu, K., Bi, J. & Tresp, V. Active learning via transductive experimental design. In *Proceedings of the 23rd international conference on Machine learning*, 1081–1088 (2006).

23. Melville, P. & Mooney, R. J. Diverse ensembles for active learning. In *Proceedings of the twenty-first international conference on Machine learning*, 74 (2004).

24. Krogh, A. & Vedelsby, J. Neural network ensembles, cross validation, and active learning. In *Advances in neural information processing systems*, 231–238 (1995).

25. Mamitsuka, N. A. H. *et al.* Query learning strategies using boosting and bagging. In *Machine learning: proceedings of the fifteenth international conference (ICML'98)*, vol. 1 (Morgan Kaufmann Pub, 1998).

26. Fedorov, V. V. *Theory of optimal experiments* (Elsevier, 2013).

27. Ingram, L. O. Ethanol tolerance in bacteria. *Critical reviews biotechnology* **9**, 305–319 (1989).

28. Maillard, J.-Y. Bacterial target sites for biocide action. *J. applied microbiology* **92**, 16S-27S (2002)..

29. Wijesinghe, L. A study on the bactericidal efficieny of selected chemical disinfectants and antiseptics. (2010).

30. Gorman, S., SCOTT, E. M. & Russell, A. Antimicrobial activity, uses and mechanism of action of glutaraldehyde. *J. Appl. Bacteriol.* **48**, 161–190 (1980).

31. Finnegan, M. *et al.* Mode of action of hydrogen peroxide and other oxidizing agents: differences between liquid and gas forms. *J. Antimicrob. Chemother.* **65**, 2108–2115 (2010).

32. DeQueiroz, G. & Day, D. Antimicrobial activity and effectiveness of a combination of sodium hypochlorite and hydrogen peroxide in killing and removing pseudomonas aeruginosa biofilms from surfaces. *J. Appl. Microbiol.* **103**, 794–802 (2007).

33. McDonnell, G. & Russell, A. D. Antiseptics and disinfectants: activity, action, and resistance. *Clin. microbiology reviews* **12**, 147–179 (1999).

34. Maris, P. Modes of action of disinfectants. *Revue scientifique et technique (International Off. Epizoot.* **14**, 47–55 (1995).

35. Selvaggi, G., Monstrey, S., Landuyt, K. V., Hamdi, M. & Blondeel, P. The role of iodine in antisepsis and wound management: a reappraisal. *Acta chirurgica belgica* **103**, 241–247 (2003).

36. Rolinson, G., Macdonald, A., Wilson, D. *et al.* Bactericidal action of β -lactam antibiotics on escherichia coli with particular reference to ampicillin and amoxycillin. *J. Antimicrob. Chemother.* **3**, 541–553 (1977).

37. Salian, S. *et al.* Structure-activity relationships among the kanamycin aminoglycosides: role of ring i hydroxyl and amino groups. *Antimicrob. agents chemotherapy* **56**, 6104–6108 (2012).

38. Shen, L., Kohlbrenner, W., Weigl, D. & Baranowski, J. Mechanism of quinolone inhibition of dna gyrase. appearance of unique norfloxacin binding sites in enzyme-dna complexes. *J. Biol. Chem.* **264**, 2973–2978 (1989).
