# Supplementary material for: Accelerated knowledge discovery from omics data by optimal experimental design
Source: Nat Commun. 2020 Oct 6;11:5026. doi: 10.1038/s41467-020-18785-y (PMC7538421; doi:10.1038/s41467-020-18785-y)
Supplement: Supplementary file 4 — Description of Additional Supplementary Files [file 41467_2020_18785_MOESM4_ESM.pdf]

Title: Supplementary Data 1:
Description: The gene expression profiles of E. coli under the 45 different culture conditions. 40 of the 45 conditions have a biocide and an antibiotic. The remaining 5 conditions include one control sample and 4 antibiotic-only samples.

Title: Supplementary Data 2:
Description: The synthetic datasets used for evaluating how OPEX is robust to noise, batch size and dataset heterogeneity.
